# Efficient Method of (*S*)-Nicotine Synthesis

**DOI:** 10.3390/molecules29235731

**Published:** 2024-12-04

**Authors:** Nazar Trotsko, Barbara Miroslaw, Radomir Jasiński, Mateusz Długosz, Małgorzata Sadczuk, Oleg M. Demchuk

**Affiliations:** 1 Department of Organic Chemistry, Medical University of Lublin, Witolda Chodźki 4A, 20-093 Lublin, Poland; 2 Department of General and Coordination Chemistry and Crystallography, Institute of Chemical Sciences, Faculty of Chemistry, Maria Curie-Sklodowska University in Lublin, Marii Skłodowskiej-Curie, 2, 20-031 Lublin, Poland; barbara.miroslaw@mail.umcs.pl; 3 Department of Organic Chemistry and Technology, University of Technology in Cracow, Warszawska 24, 31-155 Kraków, Poland; radomir.jasinski@pk.edu.pl; 4 A-Sense sp. z o.o, Przemysłowa 46, 24-320 Poniatowa, Poland; mateusz.dlugosz@a-sense.pl; 5 Chair and Department of Synthesis and Chemical Technology of Pharmaceutical Substances, Medical University of Lublin, Witolda Chodźki 4A, 20-093 Lublin, Poland; malgorzta.sadczuk@umlub.pl; 6 Faculty of Medicine, The John Paul II Catholic University of Lublin, Konstantynów 1J/4.03, 20-708 Lublin, Poland

**Keywords:** new synthetic routes for drug candidates, drug manufacturing, (*S*)-nicotine, enantiomeric separation, (*S*)-nornicotine

## Abstract

Improved methods for the synthesis of nicotine are of great importance due to the wide range of applications of synthetic nicotine, which is free from contamination with nitrosamines. Herein, we present a four-step chemical synthesis of (*S*)-nicotine, involving the reduction in myosmine, enantiomeric separation of nornicotine, and subsequent methylation of the appropriate enantiomer of nornicotine obtained. The reduction in myosmine was investigated using both electrochemical and chemical approaches, achieving up to 90% yields of pure nornicotine. To achieve the enantioseparation of nornicotine, its diastereomeric salts with chiral acids, specifically, *N*-lauroyl-(*R*)-alanine, were obtained in a mixture of methyl *tert*-butyl ether (MTBE) and chloroform, which led to the isolation of (*S*)-nornicotine with 92% *ee*. The structures of the obtained salts were determined by the X-ray diffraction (XRD) technique, which helped to explain the origin of enantiodiscrimination during the crystallization. The described methodology allows efficient regeneration of the reagents and solvents used, leading to cost-effective production of (*S*)-nicotine suitable for industrial-scale applications.

## 1. Introduction

Numerous biologically important substances containing pyrrolidine scaffold in their structure play a crucial role in medicinal chemistry [1]. A saturated sp^3^ hybridized heterocyclic scaffold constitutes a pivotal position among other pharmacophores, while the presence of the stereogenic features in it is often crucially important to ensure the efficiency of drugs and, therefore, attracts the remarkable attention of researchers [2,3,4,5].

The development of new methods for the synthesis of pyrrolidine derivatives is of great importance, particularly in the approach leading to chiral derivatives of those heterocyclic compounds. In this context, synthesizing chiral non-racemic pyrrolidine alkaloids, such as nicotine, is an excellent template for developing new synthetic strategies in this field.

Nicotine is a chiral alkaloid isolated from dried leaves of the tobacco plant *Nicotiana tabacum* [6]. *Nicotiana tabacum*, whose leaves consist of 93% of (*S*)-nicotine in a mixture of its alkaloids [7]. Several studies have demonstrated that (*S*)-nicotine and some of its analogs (e.g., altinicline) exhibited potent biological activity through modulation of nicotinic acetylcholine receptor (nAChR). (*S*)-Nicotine may have beneficial effects in the treatment of central nervous system (CNS) disorders, including Parkinson’s disease, Alzheimer’s disease, and Tourette’s syndrome [8,9,10,11]. Moreover, the altinicline (SIB-1508Y) is currently in phase II clinical trials for the treatment of Parkinson’s disease [12,13].

What is more, nicotine is widely used in nicotine replacement therapy (NRT). NRT products contain lower nicotine levels than cigarettes and lack many harmful chemicals typically present in cigarettes [14].

It has been confirmed that the (*S*)-nicotine is more biologically active than its isomer (*R*). This trend is also observed in many of its analogs. The importance of chirality for biological activity has stimulated significant efforts in synthesizing enantiopure nicotine and its derivatives.

The synthesis of (*S*)-nicotine via myosmine reduction is a well-known synthetic approach [15]. Arnold et al. [16] described a process of production of racemic nicotine involving the steps of myosmine synthesis, catalytic hydrogenation of myosmine to nornicotine, and subsequent methylation of nornicotine to (*R,S*)-nicotine. The hydrogenation of myosmine is carried out under hydrogen gas pressure ranging from atmospheric to 100 atm on 10% Pd/C catalyst in C_2_–C_3_ alcohols, alternatively, by non-catalytic reduction by sodium borohydride at room temperature.

Weber and Pan’s patent [17] relates to the preparation of enantiomerically pure (*R*)-nicotine and enantiomerically pure (*S*)-nicotine utilizing *O*,*O*’-disubstituted tartaric acids. In this method, racemic nicotine undergoes a reaction with chiral acid to form diastereomeric salts, which are separated by fractional crystallization. The resulting (*S*)-nicotine salt is treated with a base to obtain the desirable product, while the mixture enriched with the (*R*)-enantiomer is discarded as waste.

Another patent [18] claimed a method of preparation of (*S*)-nicotine using an enzyme with imine reductase activity, capable of mediating the conversion of myosmine to (*S*)-nornicotine, followed by its methylation to obtain (*S*)-nicotine. However, the availability of this enzyme is currently very limited. According to another patent [19], optically pure (*S*)-nicotine can be synthesized through asymmetric hydrogenation catalyzed by transition metal complexes. Nevertheless, there is limited evidence in the literature regarding the efficiency of these processes.

The challenges in obtaining optically pure nornicotine and the high commercial demand for its derivatives create the need for new, cost-effective processes suitable for its industrial production.

**Figure 1:**
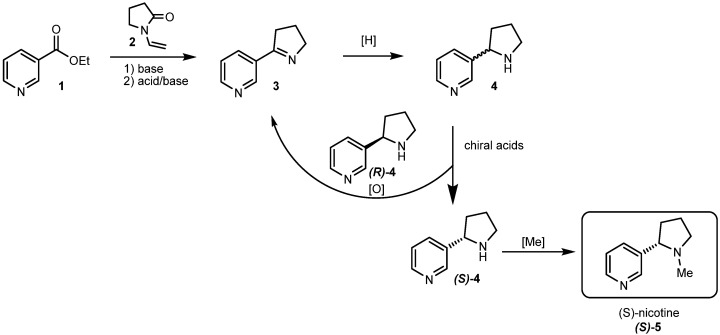
Synthetic pathway to (*S*)-nicotine (**5**) via myosmine (**3**) reduction.

This work presents an efficient synthesis of (*S*)-nicotine (**5**) involving myosmine (**3**) reduction, enantioseparation of nornicotine (**4**), and subsequent methylation of the appropriate enantiomer to (*S*)-**5** (Figure 1). Notably, in our process, the undesirable (*R*)-**4** is recovered and oxidized back to the prochiral **3**. This procedure can be performed on an industrial scale.

## 2. Results and Discussion

In general, the synthesis of enantiopure nicotine (**5**) leading through the enantioseparation of a racemate is economically justified if the undesired isomer can be utilized, preferably by configuration inversion or racemization of its stereogenic center. At the same time, the most cost-effective approach is the synthesis of nicotine carried out by the myosmine (**3**) stage, where the stereogenic center emerges at the stage of nornicotine (**4**) formation. Therefore, the following strategies should be considered beneficial: (a) asymmetric synthesis of nornicotine [17,19,20], followed by methylation of the appropriate enantiomer; (b) synthesis of racemic nornicotine, conversion of *rac*-nornicotine to *rac*-nicotine, isolation of (*S*)-nicotine from the racemic mixture [21], and conversion of the (*R*)-**5** isomer back to prochiral **3** [15]; (c) synthesis of *rac*-**4**, separation of enantiomers, conversion of (*S*)-**4** to (*S*)-**5**, and transformation of the undesired (*R*)-**4** back to prochiral **3** [22].

The analysis of possible synthetic approaches revealed the following: approach A is too expensive due to the need for fine catalysts, the high risk of non-reproducible synthesis, and obtaining of the product with low optical purity; approach B has limited practical potential because of the challenges in regenerating chiral acids used for separation and the hazardous, inefficient demethylation process of nicotine back to myosmine; approach C, implemented in our procedure, avoids these limitations due to the proven efficiency in regenerating the reagents (e.g., MnO_2_; chiral acid) and solvents used, leading to low production costs.

The design of approach C is presented in Figure 1. It includes the following steps:

(i) synthesis of **3**;
(ii) reduction in **3**;
(iii) separation of **4** enantiomers;
(iv) oxidation of the undesired (*R*)-**4** back to **3**;
(v) methylation of (*S*)-**4**.

### 2.1. Synthesis of Myosmine (***3***)

Myosmine was prepared by condensation of *N*-vinylpyrrolidone with ethyl nicotinate using a modified method previously described [17,23]. The reaction was performed in toluene in the presence of sodium methoxide. The condensation product was then treated with concentrated hydrochloric acid at a liberated temperature. After basifying with a 40% sodium hydroxide solution, myosmine was isolated by extraction and distillation, with a 60% yield.

### 2.2. Reduction in Myosmine (***3***) to Nornicotine (***4***)

The next step of synthesis was the reduction in myosmine. One of the reduction protocols was electrochemical reduction. The possibility of obtaining the desired product electrochemically was checked, and the commercial potential of this approach was assessed. Pilot reactions were carried out on a 5 mmol laboratory scale. Myosmine (5 mmol) and lithium chloride (2 mmol) were dissolved in methanol (10 mL) and electrolyzed on graphite anode and stainless steel 304 cathode with dimensions 10 mm × 40 mm × 1 mm: 100 mA, 3.3 V in an undivided cell. The reaction was carried out until the passed charge reached 2.2 F/mol. Upon completion of the electrolysis, 50% conversion of the substrate was observed (GC–MS). The reaction was repeated four times, and nornicotine was isolated from the combined reaction mixtures by fractional distillation under reduced pressure in 38%yield. 

A similar reaction, performed on both electrodes made of stainless steel 304, resulted in 98% substrate conversion. During electrolysis, the anode dissolved, resulting in the precipitation of an amorphous iron (II) salt (presumably methoxide). The iron salt was centrifuged, washed with methanol, and centrifuged again. After evaporation of methanol, nornicotine was isolated with approximately 90% purity (GC–MS). The experiment was repeated four times, and rough nornicotine fractions were combined and purified by distillation. A total of 85% yield of pure nornicotine was obtained.

Chiral additives such as D-DBTA, L-C_11_H_23_CONHCH(CH_3_)COOH, L-lactic acid, (*S*)-nicotine, fructose, lactose, L-menthol, L-limonene, as well as the replacement of LiCl with LiBF_4_, reduced the reaction efficiency and did not allow for the formation of an optically pure product. Reduction using two carbon electrodes was also ineffective due to rapid electrode degradation.

Another reduction approach to **3** was a chemical reduction. Hydrogenation attempts using a Pd/C catalyst revealed that as much as 2% by weight of 10% Pd/C catalyst was necessary to achieve good substrate conversion under 1 atm of the pressure of hydrogen at room temperature in ethanol. Attempts to reduce **3** with ammonium formate or formic acid in the presence of Pd/C did not yield a pure product, making this method economically unviable for further development.

Subsequent reactions explored the potential of **3** reduction using NaBH_4_. Due to the modest reactivity of NaBH_4_ toward imines, initially, the reactions were carried out in methanol or water or their mixture with in situ-modified borohydride by adding an acid. This approach not only accelerated the reaction, running below 0 °C but also allowed us to introduce a chiral environment to enable asymmetric reduction. The following organic and inorganic acids were tested: acetic; malic; and citric acids yielded product in 90% yield as racemate or with insufficient *ee*s, whereas the utilization of sulfuric acid and sodium hydrogen sulfate resulted in formation of racemic nornicotine in 80% yield. Ultimately, the reaction performed in methanol at ambient temperature for 48 h resulted in an almost quantitative yield of the desirable nornicotine. The extraction of the post-reaction mixture significantly impacted yield, with dichloromethane proving the most effective (but less environmentally beneficial) compared to diethyl ether or *tert*-butyl methyl ether (MTBE).

### 2.3. Separating of Nornicotine Enantiomers

Several chiral acids were selected for enantioseparation tests: D-DBTA, L-tartaric, L-malic, and L-MentOCH_2_COOH, alongside low-toxicity solvents, such as ethanol, diethyl ether, tert-butyl methyl ether, acetone, and water.

The salts of hydroxy acids (L-tartaric acid, L-malic acid) were insoluble in low polar organic solvents, requiring the use of ethanol or water for dissolution. However, under these conditions, no crystalline product was obtained. Attempts to prepare salts from nornicotine and D-DBTA in those solvents or solvent mixtures, such as acetone/ethanol, acetone/ethanol/water, and isopropanol, were also unsuccessful. Although the crystalline forms were obtained from ethanol or methanol, the enantiomeric excess (*ee*) of nornicotine was unsatisfactory, remaining below 50% (determined by NMR). Using the L-MentOCH_2_COOH in separation attempts allowed for the formation of crystalline salts in low polar aprotic solvents, such as diethyl ether or *tert*-butyl methyl ether. Unfortunately, all attempts yielded racemates.

It seems that successful enantioseparation requires reaching a subtle balance of substituents in the chiral acid, with both hydrophilic and lipophilic properties, as well as groups capable of forming additional hydrogen bonds to stabilize the crystal lattice. To address this, we prepared a small library of *N*-acyl amino acids, such as C_4_H_9_CO-L-Ala, C_4_H_9_CO-L-Phe, C_4_H_9_CO-L-Leu, C_8_H_17_CO-L-Ala, C_11_H_23_CO-L-Phe, and C_15_H_31_CO-L-Ala. However, the most favorable results of crystallization were obtained with *N*-lauroyl-(*S*)-alanine ((*S*)-**6**)) or *N*-lauroyl-(*R*)-alanine ((*R*)-**6**)), in MTBE or a mixture of MTBE and chloroform at 5:2 volume ratio. *N*-lauroyl-(*S*)-alanine and *N*-lauroyl-(*R*)-alanine were synthesized following a previously described method [24].

**Figure 2:**
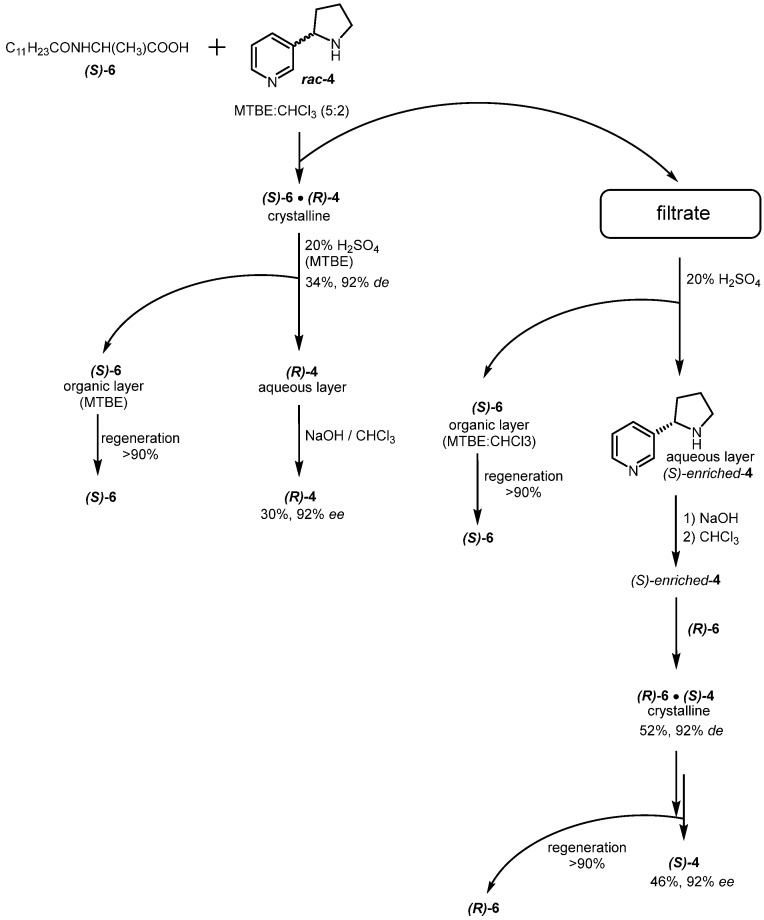
General scheme of enantioseparation of **4** enantiomers.

The enantioseparation of racemic nornicotine with the utilization of *N*-lauroyl-(*S*)-alanine or *N*-lauroyl-(*R*)-alanine proceeded as illustrated in Figure 2, with the following steps:

(a) preparation of single diastereomeric salt of (*S*)-**6**•(*R*)-**4** from *rac*-**4** by fractional crystallization;
(b) isolation of the pure stereoisomer (*R*)-**4** of nornicotine from the salt, followed by regeneration of (*S*)-**6** used;
(c) regeneration of (*S*)-**6** and **4**, enriched in isomer (*S*), from post-crystallization liquor;
(d) preparation of single diastereomeric salt (*R*)-**6**•(*S*)-**4** from (*R*)-**6** and enantioenriched **4**, obtained at the previous step;
(e) isolation of optically pure (*S*)-**4** and regeneration of (*R*)-**6.**

The utilization of *N*-lauroyl-alanines for the separation of *rac*-**4** showed optimal results in MTBE:chloroform (5:2) solvent system. The salt of (*R*)-**4** with (*S*)-**6** was obtained in 34%yield (based on a single enantiomer) and 92% diastereomeric excess (*de*). The optical purity was determined based on the measurement of the specific rotation of (*S*)-**6**•(*R*)-**4** salt or the individual (*R*)-**4**, isolated from the salts. Their *de* and *ee* values were determined based on the specific optical rotation relative to the optical rotation of the pure (*S*)-**6**•(*R*)-**4** salt, obtained in a laboratory scale as a reference compound or based on measuring the specific rotation of isolated from its salts (*R*)-**4** or (*S*)-**4** or (*S*)-**5** obtained in further methylation of (*S*)-**4**. The measurements were also confirmed by means of NMR spectroscopy, where spectra of salts (or nornicotine) were recorded in the presence of chiral discriminating agents ((+)-(*S*)-2-(6-methoxynaphthalen-2-yl)propanoic acid or (−)-*O,O′*-di-p-toluoyl-L-tartaric acid). Notably, using (*S*)-**6** yields in the crystalline product with (*R*)-**4** and using the opposite unnatural (*R*)-**6** allows us to obtain desirable natural (*S*)-**4**. This process can be accelerated (though with a slightly lower yield) when the more expensive unnatural (*R*)-**6** is initially used. On an industrial scale, the crystallization was initiated by seeding the mixture with previously obtained pure salt of appropriate nornicotine enantiomer.

The (*R*)-**4** was extracted from salts using 20% H_2_SO_4_ and MTBE. Then, after alkalizing the aqueous layer with 40% NaOH to pH = 14, nornicotine was extracted from the aqueous phase with chloroform. Chloroform was evaporated on a rotary evaporator, and the remaining water was distilled off as an azeotrope with toluene using a Dean–Stark apparatus to furnish (*R*)-**4** in 30% yield (based on a single enantiomer). Using this method, **4** enriched in (*S*)-enantiomer was isolated from the post-crystallization filtrate, with a 160% yield (based on a single enantiomer) and 20% *ee*. This enriched in (*S*)-enantiomer nornicotine was recrystallized with (*R*)-**6**, following the above procedure, to give (*S*)-**4** in 46% yield and 92% *ee*.

Importantly, (*S*)-**6** and (*R*)-**6** remaining in the organic layers were separated and regenerated in 90% yield. Another important aspect is the possibility of solvent regeneration, which significantly reduces production costs and enables the process to be conducted continuously.

### 2.4. Oxidation of Undesirable (R)-Nornicotine to Myosmine

To achieve the optimal atom economy of the process, the potential of electrochemical oxidation of the undesirable (*R*)-**4** to **3** was assessed. Reactions were conducted on a laboratory scale with 8 mmol of substrate in the presence of LiBF_4_ in 10 mL CH_3_CN. Electrolysis was carried out on Cu anode and stainless steel 304 cathode with dimensions 10 mm × 40 mm × 1 mm: 20 mA, 4V. The reaction proceeded until the total charge of 2.2 F/mol passed. During the reaction, copper transfer from the anode to the cathode was observed with the simultaneous oxidation of **4**, resulting in an 85% conversion. The experiment was repeated four times, and the combined reaction mixtures were purified by distillation. A total of 75% yield of pure **3** was obtained.

Chemical oxidation of **4** was carried out using a supra-stoichiometric amount of manganese(IV) oxide. The **4** and MnO_2_ were mixed in acetonitrile for 14 days at 30 °C, with the reaction progress monitored by GC–MS. After achieving a conversion of 95%, manganese oxide was filtered off, washed with acetonitrile, and subjected to oxidative regeneration [25]. Acetonitrile was evaporated under reduced pressure, and the obtained **3**, without purification, was converted to *rac*-**4**.

### 2.5. Methylation of (S)-***4*** to Form (S)-***5***

The pyrrolidine ring of (*S*)-**4** was methylated at the *N*-atom using formaldehyde and formic acid at 80–85 °C. The reaction was carried out in a molar ratio of 1:4:2, respectively, according to the method described earlier [20,21]. After cooling and alkalizing the post-reaction mixture, **5** was extracted and purified by vacuum distillation.

### 2.6. Crystal Structure of (S)-***6***•(R)-***4*** and (S)-***6***•(S)-***4*** Salts

**Figure 3:**
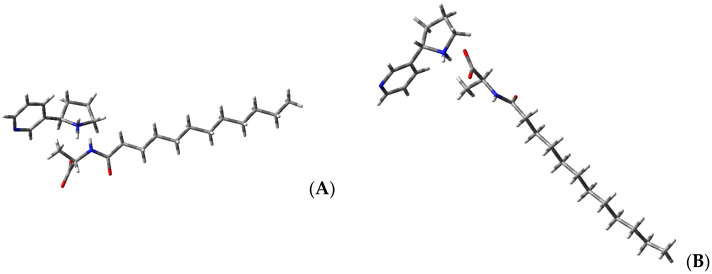
Views of the ion pairs based on the (*R*)- or (*S*)-enantiomers of nornicotine and *N*-lauroyl-(*S*)-alanine–(**A**) and (**B**), respectively.

During the crystallization, only heterochiral salts are formed ((*S*)-**6**•(*R*)-**4**); however, the homochiral salts can be obtained by crystallizing individual compounds from the MTBE/CHCl_3_ mixture. The X-ray diffraction method resolved two crystal structures of (*S*)-**6** nornicotines salts (Figure 3).

**Figure 4:**
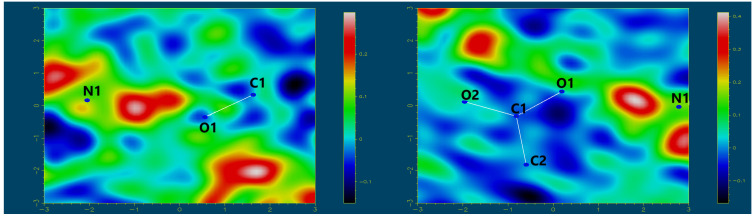
Difference Fourier maps of the electron density around the intermolecular N1–H…O1 contacts in crystals of *N*-lauroyl-(*S*)-alanine and (*R*)- (**left**) and (*S*)- (**right**) nornicotine enantiomers’ salts, indicating the electron density transfer from the carboxylate O1 towards the nornicotine N atom.

These crystals, containing two different enantiomers of nornicotine (*S-* or *R-*), crystallize in the same orthorhombic *P*2_1_2_1_2_1_ space group with nearly identical densities of 1.090 and 1.105 g/cm^3^ for (*S*)- and (*R*)- enantiomers, respectively (Figures S1 and S2, Tables S1–S4). (*S*)-**4** forms crystals only from solutions containing exclusively (*S*)- enantiomer, whereas (*R*)-**4** crystals may be obtained both from the enantiomerically pure solution of (*R*)- enantiomer and a racemic mixture. To elucidate the nature of the crystals, the difference Fourier maps of the electron density [26] around the intermolecular N1–H…O1 contacts in crystals of (*S*)- and (*R*)- enantiomers were generated (Figure 4). These maps clearly indicate the transfer of electron density from the carboxylate O1 toward the nornicotine N1 atom in both crystals, confirming the formation of salts in the solid state. 

**Figure 5:**
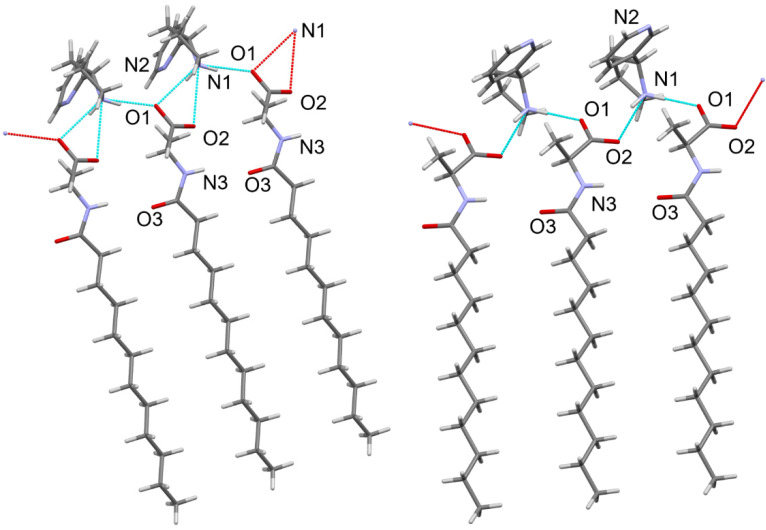
X-ray crystal packing of (*S*)-**6**•(*R*)-**4** (left) and (*S*)-**6**•(*S*)-**4** (right) enantiomers.

The number and type of potential hydrogen bond donors and acceptors are the same in both cases. The hydrogen-bonded chains in both crystals are based on two analogous N–H…O supramolecular contacts (Figure 5, Table 1). However, in the crystal of the (*R*)-**4**, a slightly different molecular arrangement allows for an additional bifurcated hydrogen bond N1–H…O2^#^ (symmetry operation: 1 + x, y, z) with the N…O distance of 2.974(5) Å. In contrast, the analogous distance in the (*S*)-**4** crystal is longer (3.122(4) Å). The amide groups in both crystals are excluded from hydrogen bonding.

The subtle structural differences are accompanied by minor energetic effects. The total packing energy calculations for both crystal structures [27,28] yielded values of −244.8 and −256.8 kJ/mol for the salts of (*S*)- and (*R*)- enantiomers, respectively, indicating a slight difference in energy, which may suggest slightly higher thermodynamic stability of the crystal with (*R*)- form. However, the interfragment energy between the amino acid and nornicotine molecules does not show a significant difference: −17.7 and −20.2 kJ/mol for (*S*)- and (*R*)-, respectively.

### 2.7. Quantumchemical Considerations

The separation of enantiomeric forms of the nornicotine using (*S*)-**6** as a co-crystallization agent can be explained based on the DFT calculations [29]. For this purpose, the m062x/6-311 + G(d) level of theory was used. The same level of theory was recently used to explain different types of energetical considerations regarding nitrogen-containing molecular systems [30,31,32]. The presence of the ethereal solution was simulated using the PCM model [33]. It was found that in the ethereal solution, the pair containing the (*S*)-**4** molecular segment exhibited the Gibbs free energy of the formation about 22 kcal/mol lower than the analogous pair based on the (*R*)-isomer. So, under the analyzed conditions, the (*S*)-**4** pair should be considered more stable from the thermodynamical point of view.

## 3. Materials and Methods

### 3.1. General

All the chemicals utilized in this research were obtained from Sigma-Aldrich (Merck KGaA, Darmstadt, Germany) and employed without further purification. The ^1^H NMR and ^13^C NMR spectra were acquired on a Bruker Avance 600 spectrometer (Bruker BioSpin GmbH, Rheinstetten, Germany) in CDCl_3_ as a solvent and TMS as an internal standard. The HPLC and MS analyses were performed on Shimadzu LCMS Q-Tof 9030 ESI (Kioto, Japan) instrument equipped with column Dr. Maisch (Dr. Maisch, Ammerbuch-Entringen, Germany) ReproSil-Pur Basic-C18 3 µm 100 mm, which was eluted at 40 °C by MeOH/H_2_O = 70/30 with rate 0.3 mL/min, the injections of analyte were applied at the first minute of elution. Specific rotation was measured at 20 °C on a Perkin Elmer 343 polarimeter (PerkinElmer, Inc., Waltham, MA, USA) equipped with a Na lamp (λ = 589 nm). The electrochemical experiments were conducted with IKA (IKA England Ltd., Oxford, UK) ElectraSyn 2.0 machine.

### 3.2. Synthesis of Myosmine (***3***)

To 50 g (0.331 mol) of ethyl nicotinate and 26.8 g (0.4965 mol) of sodium methoxide in toluene (100 mL), 40.5 g (0.364 mol) *N*-vinyl-2-pyrrolidone was added. The stirred mixture was heated under reflux for 4 h, and then it was cooled down to ambient temperature. After that, concentrated hydrochloric acid (102 mL) and water (102 mL) were added, and the mixture was heated at reflux conditions for 6 h. After cooling, 50% solution of NaOH was added to pH = 10. The mixture was then extracted with toluene (3 × 100 mL). The extract was dried over MgSO_4_, and the solvent was evaporated under reduced pressure. The residue was purified by vacuum distillation to obtain 27.5 g (57%) of **3**. M.p. = 41.0–42.2 °C. ^1^H NMR (600 MHz, CDCl_3_) δ (ppm): 1.99–2.06 (m, 2 H), 2.90–2,94 (m, 2 H), 4.04 (tt, *J =* 7.4, 2.0), 7.30 (dd, *J =* 8.0, 4.0, 1 H), 8.14 (dt, *J =* 8.0, 2.0, 1 H), 8.61 (dd, *J =* 5.2, 1.7, 1 H), 8.95 (d, *J =* 2.3, 1 H); ^13^C NMR (150 MHz, CDCl_3_) δ (ppm): 22.3, 34.6, 61.5, 123.3, 130.1, 134.5, 149.0, 151.1, 170.9.

### 3.3. Synthesis of Racemic Nornicotine (***4***)

#### 3.3.1. Chemical Synthesis of *rac*-**4**

A total of 20 g (0.137 mol) of **3** was dissolved in 450 mL of methanol and 150 mL of water. The stirred solution was cooled to 15 °C, and NaBH_4_ (7 g, 0.185 mol) was added (1 g every 20 min). The reaction mixture was kept for 12 h at 15 °C, followed by an additional 12 h at room temperature. After 24 h, the reaction mixture was checked for the presence of myosmine using the GC–MS method. Maximum myosmine content in the reaction mixture should be 2–3%. Methanol was then evaporated under reduced pressure. The reaction mixture was alkalized by 40% NaOH to pH = 14 and extracted with MTBE (4 × 150 mL). The combined organic layer was dried over MgSO_4_. MTBE was evaporated under reduced pressure. The residue was purified by vacuum distillation (1 mbar, 135–140 °C) to obtain 17.9 g (88%) of **4**. ^1^H NMR (600 MHz, CDCl_3_) δ (ppm): 1.61–1.69 (m, 1 H), 1.81–1.97 (m, 2 H), 2.08 (bs, 1 H), 2.17–2.24 (m, 1 H), 3.03 (ddd, *J =* 10.1, 8.2, 6.6, 1 H), 3.18 (ddd, *J =* 10.1, 7.7, 5.2, 1 H), 4.14 (t, *J =* 7.7, 1 H), 7.21–7.24 (m, 1 H), 7.68–7.71 (m, 1 H), 8.46 (dd, *J =* 4.9, 1.7, 1 H), 8.58 (d, *J =* 2.0, 1 H); ^13^C NMR (150 MHz, CDCl_3_) δ (ppm): 25.6, 34.4, 47.0, 60.1, 123.4, 134.1, 140.4, 148.4, 148.7.

#### 3.3.2. Electrochemical Synthesis of *rac*-**4**

In the standard IKA undivided electrochemical cell equipped with two stainless still 304 electrodes, a solution of **3** (670 mg, 5 mmol), lithium chloride (90 mg, 2 mmol) in 10 mL of methanol was placed. The IKA (IKA England Ltd., Oxford, UK) ElectraSyn 2.0 machine was set up on 100 mA (3.3 V; during the reaction, the voltage is changed), 2.2 F/mol. The time of the reaction run is controlled by the ElectraSyn 2.0 machine and is about several hours. After the completion of the reaction time, the reaction mixture was centrifuged to separate the solution from iron salt, and methanol was then evaporated under reduced pressure. The residue was basified with 10 mL of 30% sodium hydroxide, and the product was extracted with TBME (3 × 20 mL). The organic phase was analyzed by GC–MS. After the repetition of the experiment 4 times, the combined extracts were dried over the MgSO_4_, and **4** was isolated by distillation under the reduced pressure to obtain 2.3 g (85%) of above 98% purity.

### 3.4. Separating of Enantiomers of ***4***

#### 3.4.1. Preparation of *N*-Lauroyl-(*S*)-Alanine•(*R*)-Nornicotine Salt

To a solution of 373.17 g (1.377 mol) (*S*)-**6** in 1285 mL MTBE and 515 mL chloroform (5:2), 203.8 g (1.377 mol) of *rac*-**4** was added. The solution was brought to a boil and allowed to cool slowly. When the temperature was lowered to 30 °C, the solution was seeded with the previously synthesized (*S*)-**6**•(*R*)-**4**, adding small portions of salt every 3 °C. Once crystallization began, the mixture was stirred and left to crystallize at room temperature. The precipitate was then filtered under reduced pressure, recrystallized from the minimal amount of MTBE/CHCl_3_ mixture, and dried, yielding 196.17 g (34%), 92% *de*, [α]^20^_D_ = −7.8 (c 1, MeOH). The higher *de* can be achieved after additional recrystallization of the obtained salt from the same solvent system.

M.p. = 79–80 °C. ^1^H NMR (600 MHz, CDCl_3_) δ (ppm): 0.89 (t, *J* = 7.2 Hz, 3 H); 1.26–1.30 (m, 20 H); 1.57–1.60 (m, 2 H); 2.07–2.12 (m, 1 H); 2.16 (t, *J* = 7.2 Hz, 2 H); 2.18–2.23 (m, 2 H); 2.38–2.42 (m, 1 H); 3.22–3.26 (m, 1 H); 3.31–3.36 (m, 1 H); 4.19 (quint, *J* = 7.2 Hz, 1 H); 4.50 (dd, *J* = 7.2, 10.2 Hz, 1 H); 6.48 (d, *J* = 6.6 Hz, 1 H); 7.31 (dd, *J* = 4.8, 8.4 Hz, 1 H); 7.88 (dt, *J* = 1.8, 7.8 Hz, 1 H); 8.56 (dd, *J* = 1.8, 4.8 Hz, 1 H); 8.66 (d, *J* = 2.4 Hz, 1 H); 9.37 (bs, 2 H). ^13^C NMR (150 MHz, CDCl_3_) δ (ppm): 14.1, 18.9, 18.9, 22.7, 23.7, 25.8, 29.3, 29.6, 31.5, 31.9, 36.7, 44.8, 49.8, 49.9, 60.4, 123.8, 131.4, 135.5, 135.6, 149.2, 149.2, 150.1, 172.9, 177.8.

#### 3.4.2. Isolation of (*R*)-**4** and Recovery of (*S*)-**6** from (*S*)-**6**•(*R*)-**4** Salt

To 196.17 g (0.468 mol) (*S*)-**6***•*(*R*)-**4**, 300 mL MTBE and 250 mL of 20% sulfuric acid were added, resulting in pH = 2. The organic layer was separated and dried over MgSO_4_. After the evaporation of MTBE, (*S*)-**6** was dried under reduced pressure (yield 114.12 g, 90%). The aqueous layer was then alkalized by 140 g of 50% NaOH to pH = 14. Next, (*R*)-**4** was extracted with chloroform (2 × 130 mL). Chloroform was evaporated under reduced pressure, and residual water was removed by azeotropic distillation with the Dean–Stark apparatus. The (*R*)-**4** was purified by vacuum distillation (3.8 mbar temp. 108 °C or 1.8 mbar temp. 88 °C).

Yield 54.3 g (78%), [α]^20^_D_ = + 29.9 (c 1, MeOH), 92% *ee*.

^1.^ H NMR (600 MHz, CDCl_3_) δ (ppm): 1.51–1.57 (m, 1 H); 1.71–1.85 (m, 2 H); 2.07–2.12 (m, 1 H); 2.58 (s, 1 H); 2.92 (ddd, 1 H, *J* = 10.2, 8.3, 6.7 Hz); 3.06 (ddd, 1 H, *J* = 10.2, 7.7, 5.3 Hz); 4.03 (t, 1 H, *J* = 7.8 Hz); 7.11 (ddd, 1 H, *J* = 7.9, 4.8, 0.9 Hz); 7.60 (dt, 1 H, *J* = 7.8, 2.1 Hz); 8.34 (dt, 1 H, *J* = 4.8, 1.5 Hz); 8.46 (d, 1 H, *J* = 2.3 Hz). ^13^ C NMR (150 MHz, CDCl_3_) δ (ppm): 25.4, 34.2, 46.8, 59.9, 123.3, 134.0, 140.0, 148.1, 148.5.

#### 3.4.3. Recovery of (*S*)-**6** and **4** from Filtrate

To the post-crystallization filtrate, 350 mL of 20% sulfuric acid was added to adjust the pH to 2. The organic layer was separated and dried over MgSO_4_. After the evaporation of organic solvents, (*S*)-**6** was dried under reduced pressure (yield 221.6 g (90%)). The aqueous layer was alkalized by 185 g of 50% NaOH to pH = 14. Next, **4** was extracted with chloroform (2 × 200 mL). Chloroform was evaporated under reduced pressure. To the residue, toluene was added and distilled with Dean–Stark apparatus to remove water. The nornicotine was purified by vacuum distillation (3.8 mbar temp. 108 °C), yielding 111.6 g (83%). The recovered **4**, enriched in (*S*)-enantiomer, was used in the next cycle of nornicotine enantiomers separation.

#### 3.4.4. Preparation of (*R*)-**6**•(*S*)-**4**

Preparation of (*R*)-**6**•(*S*)-**4** was performed according to 3.4.1. using 204.34 g (0.754 mol) of (*R*)-**6** and 111.6 g (0.754 mol) of **4** enriched in (*S*)-enantiomer and solvents MTBE (715 mL) and chloroform (285 mL). Yield 164.2 g (52%), 92 *de*, [α]^20^_D_ = +7.7 (c 1, MeOH). A higher *de* can be achieved by recrystallizing the obtained salt from the same solvent system.

M.p. = 79–80 °C. ^1^ H NMR (600 MHz, CDCl_3_) δ (ppm): 0.89 (t, *J* = 7.1 Hz, 3 H); 1.27–1.29 (m, 18 H); 1.41 (d, *J* = 7.1 Hz, 3 H); 1.61 (quint, *J* = 7.6 Hz, 2 H); 2.00–2.08 (m, 1 H); 2.20 (dd, *J* = 7.0, 8.2 Hz, 2 H); 2.23–2.30 (m, 1 H); 2.40–2.47 (m, 1 H); 3.32–3.37 (m, 1 H); 3.45–3.50 (m, 1 H); 4.44 (quint, *J* = 7.1 Hz, 1 H); 4.57 (dd, *J* = 6.6, 10.4 Hz, 1 H); 6.43 (d, *J* = 7.0 Hz, 1 H); 7.38 (dd, *J* = 4.7, 8.2 Hz, 1 H); 8.06 (dt, *J* = 1.9, 8.0 Hz, 1 H); 8.58 (dd, *J* = 1.8, 4.7 Hz, 1 H); 8.73 (d, *J* = 2.0 Hz, 1 H); 9.74 (bs, 2 H).

^13.^ C NMR (150 MHz, CDCl_3_) δ (ppm): 14.1, 18.4, 22.7, 23.7, 25.7, 29.3, 29.4, 29.4, 29.5, 29.6, 31.2, 31.9, 36.6, 45.0, 48.8, 60.8, 123.9, 124.1, 130.6, 136.5, 148.8, 149.8, 173.4, 176.1.

#### 3.4.5. Isolation of (*S*)-**4** and Recovery of (*R*)-**6** from (*R*)-**6**•(*S*)-**4**

Isolation of (*S*)-**4** and recovery of (*R*)-**6** were performed according to the procedure in 3.4.2. The yield of (*R*)-**6** was 97.5 g (91%).

The yield of (*S*)-**4** was 45.8 g (79%), [α]^20^_D_ = -29.8 (c 1, MeOH), 91% *ee*.

^1.^ H NMR (600 MHz, CDCl_3_) δ (ppm): 1.57–1.64 (m, 1 H); 1.77–1.91 (m, 2 H); 2.13–2.18 (m, 1 H); 2.81 (s, 1 H); 2.96–3.00 (m, 1 H); 3.12 (ddd, 1 H, *J* = 10.4, 7.8, 5.3 Hz); 4.09 (t, 1 H, *J* = 7.8 Hz); 7.17 (ddd, 1 H, *J* = 7.2, 4.8, 2.0 Hz); 7.66 (dt, 1 H, *J* = 8.0, 2.1 Hz); 8.39 (dt, 1 H, *J* = 4.4, 2.1 Hz); 8.51 (d, 1 H, *J* = 2.2 Hz). ^13^C NMR (150 MHz, CDCl_3_) δ (ppm): 25.4, 34.2, 46.8, 60.0, 123.3, 134.1, 139.8, 148.2, 148.5.

#### 3.4.6. Recovery of (*R*)-**6** and Nornicotine from Filtrate

Recovery of (*R*)-**6** and nornicotine was carried out according to 3.4.3. Yield 89.3 g (91%) of (*R*)-**6** and 32.7 g (61%) **4** enriched in (*R*)-enantiomer.

#### 3.4.7. Determination of *de* and *ee* Values by Optical Rotation Power

Values of *de* and *ee* were calculated based on the specific optical rotation relative to the optical rotation of the pure (*S*)-**6***•*(*R*)-**4** or (*R*)-**6***•*(*S*)-**4** obtained on a laboratory scale (from optically pure commercial nornicotine) or based on measuring the specific rotation of isolated from their salts (*R*)-**4** or (*S*)-**4**. The enantiomeric excess of (*S*)-**5** was calculated based on European Pharmacopeia. The specific rotations of enantiomerically pure (*S*)-**6***•*(*R*)-**4** salt [α]^20^_D_ = −8.5 (c 1, MeOH), (*R*)-**6***•*(*S*)-**4** salt [α]^20^_D_ = +8.4 (c 1, MeOH), (*R*)-**4** [α]^20^_D_ = +32.8 (c 1, MeOH), (*S*)-**4** [α]^20^_D_ = −32.6 (c 1, MeOH). 

#### 3.4.8. Determination of Enantiomeric Excess of **4** by NMR

A total of 14,82 mg of **4** was weighed directly into the NMR tube, followed by the addition of 39 mg of Tol-DBTA and 0.7 mL of CDCl_3_. The NMR tube was closed with a PP cap and heated at about 100 °C until the complete dissolution of all components. The ^13^C and ^1^H NMR spectra were recorded as soon as the sample solution was cooled down to rt. The integration of signals, which are split, corresponds to the enantiomerical composition of **4**.

Alternatively, 7 mg of compound **4** (**5** or their salts with **6**) was weighed directly into the NMR tube, followed by the addition of 60 mg of Naproxen and 0.7 mL of CDCl_3_. The NMR tube was sealed with a PP cap and heated at about 100 °C until the complete dissolution of all components. The ^13^C and ^1^H NMR spectra were recorded as soon as the sample solution was cooled down to rt. The integration of signals, which are splits (aromatic hydrogens and aromatic and aliphatic carbons), corresponds to the enantiomerical composition of tested compounds [34].

### 3.5. Oxidation of Undesirable (R)-***4*** to ***3***

#### 3.5.1. Chemical Oxidation of Undesirable (*R*)-**4** to **3**

A total of 10 g of **4** and 10 g of MnO_2_ were stirred in 20 mL of CH_3_CN for 14 days at 30 °C, with the reaction progress monitored by GC–MS. After achieving a conversion of 95%, manganese oxide was filtered off, washed with acetonitrile, and subjected to oxidative regeneration. Acetonitrile was evaporated under reduced pressure, yielding **3** (6.9 g), which was then converted to *rac*-**4**, as described above, without preliminary purification.

#### 3.5.2. Electrochemical Oxidation of Undesirable (*R*)-**4** to **3**

To the standard IKA undivided electrochemical cell equipped in scarified copper anode and stainless still 304 cathodes, a solution of **4** (1.2 g, 8 mmol) and lithium tetrafluoroborate (200 mg, 2 mmol) in 10 mL of acetonitrile was added. The IKA (IKA England Ltd., Oxford, UK) ElectraSyn 2.0 machine was set up on 20 mA (4 V as an initial parameter), 2.2 F/mol. The time of the reaction run was controlled by the ElectraSyn 2.0 machine and was about several hours. After the completion of the reaction time, the solvent was evaporated under reduced pressure, the residue was basified with 10 mL of 30% sodium hydroxide, and the product was extracted with TBME (3 × 20 mL). The organic phase was analyzed by GC–MS. After the repetition of the experiment 4 times, the combined extracts were dried over the MgSO_4_, and **3** was isolated by distillation under the reduced pressure to obtain 0.9 g (75%) of 95% purity.

### 3.6. Preparation of (S)-***5***

To a stirred solution of (*S*)-**4** (7.88 g 0.053 mol) in 50 mL of water, a mixture of 37% formaldehyde (6.36 g 0.212 mol) and 85% formic acid (4.88 g 0.106 mol) was added, and the reaction was carried out at 80–85 °C for 20 h. The reaction mixture was then cooled, and pH was adjusted to 13 using 40% NaOH. Then, the product was extracted with DCM (2 × 25 mL), dried over MgSO_4_, and the solvent was removed completely. The residue was purified by vacuum distillation, yielding 8.1 g (94%) of (*S*)-**5**, [α]^20^_D_ = −135.6 (c 1, MeOH), 91% *ee*. Scaling up the reaction to an industrial scale (5 kg of (*S*)-**5**) produced similar results.

^1^H NMR (600 MHz, CDCl_3_) δ (ppm): 1.64–1.78 (m, 2 H); 1.90 (tdt, 1 H, *J* = 17.5, 9.3, 4.0 Hz); 2.10 (s, 3 H); 2.11–2.16 (m, 1 H); 2.26 (q, 1 H, *J* = 9.2 Hz); 3.04 (t, 1 H, *J* = 8.4 Hz); 3.17–3.21 (m, 1 H); 7.18 (dd, 1 H, *J* = 7.9, 4.8 Hz); 7.65 (d, 1 H, *J* = 7.8 Hz); 8.42 (dd, 1 H, *J* = 4.8, 1.7 Hz); 8.46 (d, 1 H, *J* = 2.1 Hz). ^13^ C NMR (150 MHz, CDCl_3_) δ (ppm): 22.5, 35.0, 40.2, 56.9, 68.9, 123.6, 134.8, 138.3, 148.7, 149.5.

### 3.7. X-Ray Diffraction

Diffraction intensities were measured at room temperature from single crystals of *N*-lauroyl-(*S*)-alanine (*S*)- and (*R*)-nornicotine salts on a SuperNova diffractometer with AtlasS2 detector (SuperNova, Rigaku, Osaka, Japan). The structures were solved using Olex2-1.5 [35], with the SHELXT [36] via Intrinsic Phasing, and refined with the SHELXL refinement package. The absolute configuration of nornicotine was established based on a chiral reference molecule of known absolute configuration (*N*-lauroyl-(*S*)-alanine). The H atoms attached to amide N3 and nornicotine N1 atoms were found in the Fourier difference electron density map and were refined isotropically. All other H atoms were positioned geometrically and refined by a riding model (U_iso_ 1.2). The difference Fourier maps of the electron density for crystals were generated with the use of MAPVIEW WinGX v1.80.05 [26]. The crystal structure refinement and molecular geometry data can be found in the Supplement (Tables S1–S4).

Crystal Data of *N*-lauroyl-(*S*)-alanine•(*S*)-nornicotine salt C_24_H_41_N_3_O_3_ (*M* =419.60 g/mol): orthorhombic, space group *P*2_1_2_1_2_1_ (no. 19), *a* = 5.3833(3) Å, *b* = 12.4329(10) Å, *c* = 38.203(3) Å, *V* = 2556.9(3) Å^3^, *Z* = 4, *T* = 293 K, μ(Cu Kα) = 0.566 mm^−1^, *D_calc_* = 1.090 g/cm^3^, 17,375 reflections measured (7.478° ≤ 2Θ ≤ 161.23°), and 5236 unique (*R*_int_ = 0.0361, *R*_sigma_ = 0.0365) were used in all calculations. The final *R*_1_ was 0.0554 (I > 2σ(I)), and *wR*_2_ was 0.1718 (all data). CCDC No. 2377057.

Crystal Data of *N*-lauroyl-(*S*)-alanine•(*R*)-nornicotine salt C_24_H_41_N_3_O_3_ (*M* =419.60 g/mol): orthorhombic, space group *P*2_1_2_1_2_1_ (no. 19), *a* = 5.0002(2) Å, *b* = 12.6394(7) Å, *c* = 39.897(2) Å, *V* = 2521.4(2) Å^3^, *Z* = 4, *T* = 293 K, μ(Cu Kα) = 0.574 mm^−1^, *D_calc_* = 1.105 g/cm^3^, 6729 reflections measured (7.336° ≤ 2Θ ≤ 152.022°), and 4262 unique (*R*_int_ = 0.0196, *R*_sigma_ = 0.0367) were used in all calculations. The final *R*_1_ was 0.0575 (I > 2σ(I)), and *wR*_2_ was 0.1707 (all data). CCDC No. 2377056.

### 3.8. Total Packing Energies Calculations

The total packing energies were calculated using the UNI Intermolecular Potentials implemented in Mercury 4.0 CSD software [27,28,37] based on approximate empirical pair-potential parameters, including hydrogen bonds and other interactions, such as aromatic, etc. The initial structures were taken from the XRD structures.

### 3.9. DFT Calculations

The DFT calculations were performed based on the m062x/6-311 + G(d) level of theory using the Gaussian 16, Revision C.01 software package [38]. The PlGrid infrastructure (“Ares” cluster) in the computing center “Cyfronet” was applied (grant no. PLG/2023/016326). It was established that this level of theory was adequate for the estimation of the thermodynamical stability of organic molecules [32,39]. The vibrational analysis of model molecules was performed using the FREQ keyword according to standard procedures [31].

## 4. Conclusions

The presented approach to the production of chiral non-racemic nicotine via myosmine reduction, enantioseparation of nornicotine isomers by fractional crystallization with *N*-lauroylalanines, and subsequent methylation to obtain an appropriate enantiomer of nicotine of excellent optical purity offers several advantages: high yield and atom economy; simple regeneration of crucial reagents (e.g., MnO_2_, chiral acids, undesirable isomer of nornicotine) and solvents; accessible and safe reaction conditions; and generally low production costs. The efficient regeneration of the undesired nornicotine enantiomer by its oxidation back to myosmine facilitates a closed-loop synthesis process and reduces waste. That makes our approach economically viable. Additionally, we indicate that key reaction steps could be realized electrochemically, though this requires additional studies and an efficient engineering setup.

Based on the X-ray diffraction analysis of both isolated diastereomeric *N*-lauroyl-(*S*)-alanine•(*S*)-nornicotine and *N*-lauroyl-(*S*)-alanine•(*R*)-nornicotine salts, which revealed structural differences in the crystals, as well as advanced quantum chemical calculations, we were able to explain the crystallization behavior of (*S*)- and (*R*)-nornicotine. The subtle structural differences observed in the diastereomers are accompanied by small energetic effects, which may suggest a slightly higher thermodynamic stability of the crystalline salt with (*R*)-nornicotine. However, the interfragment energy between the amino acid and nornicotine molecules does not show a significant difference.

At the same time, crystals of (*S*)-nornicotine exhibit shorter and more ordered hydrogen bonds, requiring more time to form and making the organization of lauric acid’s aliphatic chains more challenging. In contrast, (*R*)-nornicotine forms longer hydrogen bonds more quickly, allowing for its aliphatic chains sufficient space to obtain better reorganization within a denser crystal lattice. Thus, the crystallization of the salt with only the (*R*)-nornicotine enantiomer from the racemic mixture may be attributed to subtle packing effects during nucleation and crystal growth, resulting in slightly denser crystals. The (*R*)-nornicotine exhibits a stronger tendency for rapid organization in the crystalline form. Since the crystal growth is rather kinetically controlled, the formation of *N*-lauroyl-(*S*)-alanine•(*R*)-nornicotine is preferred.

The obtained results suggest that the described method can be successfully applied on an industrial scale for the production of optically pure (*S*)-nicotine, which is of considerable importance for pharmaceutical and therapeutic applications.

## Figures and Tables

**Table 1:** Selected molecular geometry and intermolecular interaction in crystal of *N*-lauroyl-(*S*)-alanine salts of (*S*)- and (*R*)-nornicotine enantiomers [Å, °].

Crystal	D–H…A	D–H	H…A	D…A	<DHA	Symm. Operation
(*S*)-**6**•(*S*)-**4**	N1–H1a…O1	0.97	1.67	2.634(4)	172	–
	N1–H1b…O2^#^	0.97	1.86	2.783(4)	158	1 + x, y, z
(*S*)-**6**•(*R*)-**4**	N1–H1b…O1	1.09	1.61	2.659(6)	159	–
	N1–H1a…O1^#^	0.81	2.07	2.860(5)	166	1 + x, y, z
	N1–H1a…O2^#^	0.81	2.54	2.974(5)	115	1 + x, y, z

## Data Availability

Data are contained within the article and supplementary materials.

## Supplementary Materials

The following supporting information can be downloaded at https://www.mdpi.com/article/10.3390/molecules29235731/s1. Figures S1 and S2. Molecular structure of *N*-lauroyl-(*S*)-alanine (S)- and (*R*)-nornicotine salt with atom numbering scheme. Table S1 Crystal data and structure refinement for *N*-lauroyl-(S)-alanine nornicotine salts. Table S2 Bond Lengths for *N*-lauroyl-(*S*)-alanine nornicotine salts. Table S3 Bond Angles for N-lauroyl-(S)-alanine nornicotine salts. Table S4 Torsion Angles for *N*-lauroyl-(*S*)-alanine nornicotine salts. Figures S3 and S4. ^1^H and ^13^C NMR spectra of *N*-lauroyl-(S)-alanine (*R*)-nornicotine salt. Figures S5 and S6. ^1^H and ^13^C NMR spectra of *N*-lauroyl-(*R*)-alanine (*S*)-nornicotine salt. Figures S7 and S8. ^1^H and ^13^C NMR spectra of (*R*)-nornicotine. Figures S9 and S10. ^1^H and ^13^C NMR spectra of (*S*)-nornicotine. Figures S11 and S12. ^1^H and ^13^C NMR spectra of (*S*)-nicotine. Figures S13 and S14. ^1^H and ^13^C NMR spectra of myosmine. Figure S15. HRMS analysis of myosmine (Q-Tof). Figure S16. HRMS analysis of nornicotine (Q-Tof). Figure S17. HRMS analysis of nicotine (Q-Tof). Figure S18. GCMS analysis of reaction mixture of incomplete oxidation of nornicotine to myosmine. Figure S19. GCMS analysis of reaction mixture of incomplete reduction in myosmine. Figure S20. GCMS analysis of nicotine. Figure S21. Comparision of DEPT135 spectra of *rac*-nornicotine and *rac*-nornicotine in the presence of naproxen. Figure S22. Comparision of ^1^H NMR spectra of *rac*-nornicotine and *rac*-nornicotine in the presence of naproxen. Crystallographic data for the structures of N-lauroyl-(*S*)-alanine (*R*)- and (*S*)-nornicotine salts have been deposited with the Cambridge Crystallographic Data Centre as supplementary publication no. CCDC 2377056 and 2377057. Copies of the data can be obtained, free of charge, on application to CCDC, 12 Union Road, Cambridge, CB121EZ, UK. Fax: +44 1223 366 033, e-mail: deposit@ccdc.ac.uk or on the web http://www.ccdc.cam.ac.uk.
