# Supplementary material for: Efficient Method of (S)-Nicotine Synthesis
Source: Molecules. 2024 Dec 4;29(23):5731. doi: 10.3390/molecules29235731 (PMC11643282; doi:10.3390/molecules29235731)
Supplement: Supplementary file 1 [file molecules-29-05731-s001.zip › molecules-3300587-supplementary.pdf]

molecules

MDPI

Supplementary Materials

Efficient Method of (*S*)-Nicotine Synthesis

Nazar Trotsko[1]*, Barbara Miroslaw[2], Radomir Jasiński[3], Mateusz Długosz[4], Małgorzata Sadczuk[5] and Oleg M. Demchuk[6]*

[1] Department of Organic Chemistry, Medical University of Lublin, Witolda Chodźki 4A, 20-093 Lublin, Poland
[2] Department of General and Coordination Chemistry and Crystallography, Institute of Chemical Sciences, Faculty of Chemistry, Maria Curie-Sklodowska University in Lublin, Marii Skłodowskiej-Curie, 2, 20-031 Lublin, Poland, barbara.miroslaw@mail.umcs.pl
[3] Department of Organic Chemistry and Technology, University of Technology in Cracow, Warszawska 24, 31-155 Kraków, Poland, radomir.jasinski@pk.edu.pl
[4] A-Sense sp. z o.o, Przemysłowa 46, 24-320 Poniatowa, Poland, mateusz.dlugosz@a-sense.pl
[5] Chair and Department of Synthesis and Chemical Technology of Pharmaceutical Substances, Medical Univer-sity of Lublin, Witolda Chodźki 4A, 20-093 Lublin, Poland; malgorzta.sadczuk@umlub.pl
[6] Faculty of Medicine, The John Paul II Catholic University of Lublin, Konstantynów 1J/4.03, 20-708 Lublin, Poland

* Correspondence: nazar.trotsko@umlub.pl (N.T.); Oleh.Demchuk@KUL.Lublin.pl (O.D.)

Table of contents:

Figure S1. Molecular structure of *N*-lauroyl-(*S*)-alanine (*S*)-nornicotine salt with atom numbering scheme 2
Figure S2. Molecular structure of *N*-lauroyl-(*S*)-alanine (*R*)-nornicotine salt with atom numbering scheme 2
Table S1 Crystal data and structure refinement for *N*-lauroyl-*S*-alanine (*S*)- and (*R*)-nornicotine salts 3
Table S2 Bond Lengths for *N*-lauroyl-S-alanine (*S*)- and (*R*)-nornicotine salts 4
Table S3 Bond Angles for *N*-lauroyl-*S*-alanine (*S*)- and (*R*)-nornicotine salts 5
Table S4 Torsion Angles for *N*-lauroyl-*S*-alanine (*S*)- and (*R*)-nornicotine salts 6
Figures S3 and S4. ^{1}H and ^{13}C NMR spectra of *N*-lauroyl-(*S*)-alanine (*R*)-nornicotine salt 7
Figures S5 and S6. ^{1}H and ^{13}C NMR spectra of *N*-lauroyl-(*R*)-alanine (*S*)-nornicotine salt 8
Figure S7 and S8. ^{1}H and ^{13}C NMR spectra of (*R*)-nornicotine 9
Figures S9 and S10. ^{1}H and ^{13}C NMR spectra of (*S*)-nornicotine 10
Figures S11 and S12. ^{1}H and ^{13}C NMR spectra of (*S*)-nicotine 11
Figure S13 and S14. ^{1}H and ^{13}C NMR spectra of myosmine 12
Figure S15. HRMS analysis of myosmine (Q-Tof) 15
Figure S16. HRMS analysis of nornicotine (Q-Tof) 14
Figure S17. HRMS analysis of nicotine (Q-Tof) 15
Figure S18. GCMS analysis of reaction mixture of incomplete oxidation of nornicotine to myosmine 16
Figure S19. GCMS analysis of reaction mixture of incomplete reduction of myosmine 17
Figure S20. GCMS analysis of nicotine 18
Figure S21. Comparision of DEPT135 spectra of rac-nornicotine and rac-nornicotine in the presence of naproxen 19
Figure S22. Comparision of ^{1}H NMR spectra of rac-nornicotine and rac-nornicotine in the presence of naproxen 20

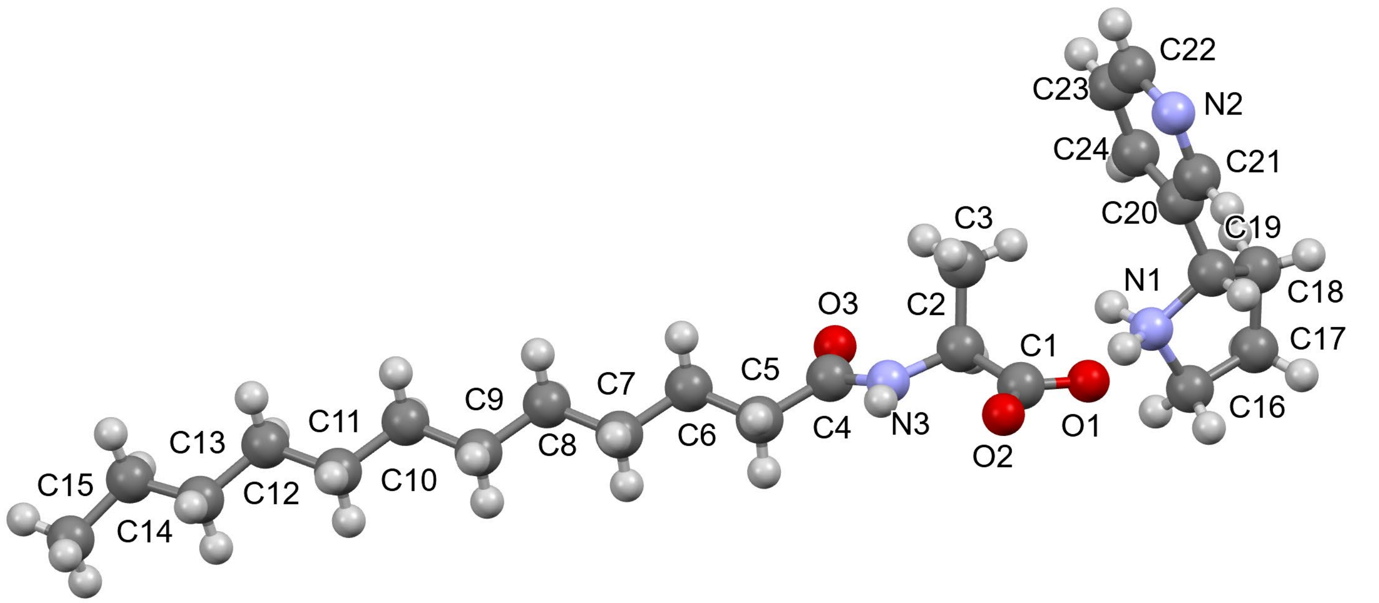

Figure S1. Molecular structure of *N*-lauroyl-*(S)*-alanine *(S)*-nornicotine salt with atom numbering scheme.

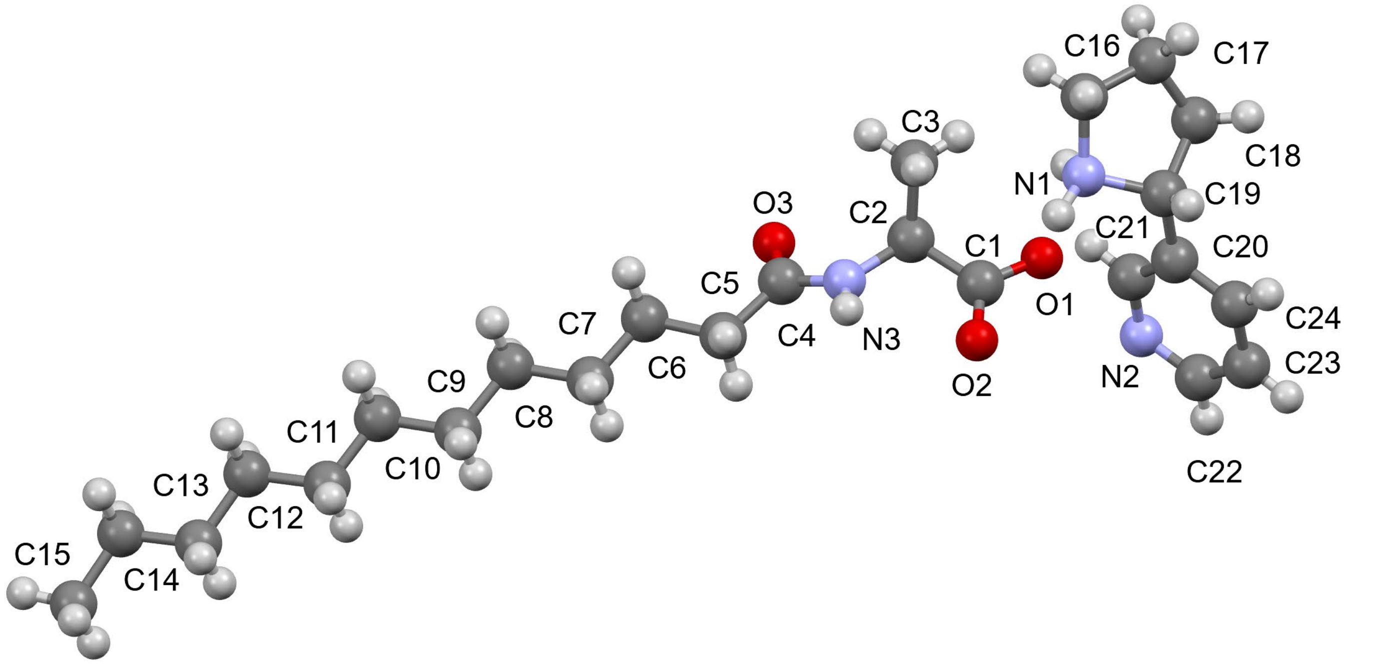

Figure S2. Molecular structure of *N*-lauroyl-*(S)*-alanine *(R)*-nornicotine salt with atom numbering scheme.

Table S1 Crystal data and structure refinement for *N*-lauroyl-*(S)*-alanine *(S)*- and *(R)*- nornicotine salts.

Identification code	*N*-lauroyl-(*S)*-alanine (*S*)-nornicotine	*N*-lauroyl-(*S)*-alanine (*R*)-nornicotine
Empirical formula	$C_{24}H_{41}N_3O_3$	$C_{24}H_{41}N_3O_3$
Formula weight	419.60	419.60
Temperature/K	293	293
Crystal system	orthorhombic	orthorhombic
Space group	$P2_12_12_1$	$P2_12_12_1$
a/Å	5.3833(3)	5.0002(2)
b/Å	12.433(1)	12.6394(7)
c/Å	38.203(3)	39.897(2)
α/°	90	90
β/°	90	90
γ/°	90	90
Volume/Å^3	2556.9(3)	2521.4(2)
Z	4	4
ρ_{calc} g/cm^3	1.090	1.105
μ/mm^{-1}	0.566	0.574
F(000)	920.0	920.0
Crystal size/mm^3	0.25 × 0.1 × 0.05	0.25 × 0.08 × 0.05
Radiation	Cu Kα (λ = 1.54184)	Cu Kα (λ = 1.54184)
2Θ range for data collection/°	7.478 to 161.23	7.336 to 152.022
Index ranges	$-6 \le h \le 6$, $-14 \le k \le 15$, $-44 \le l \le 47$	$-5 \le h \le 6$, $-13 \le k \le 15$, $-49 \le l \le 47$
Reflections collected	17375	6729
Independent reflections	5236 [R_{int} = 0.0361, R_{sigma} = 0.0365]	4262 [R_{int} = 0.0196, R_{sigma} = 0.0367]
Data/restraints/parameters	5236/0/285	4262/0/285
Goodness-of-fit on F^2	1.119	1.034
Final R indexes [I>=2σ(I)]	R_1 = 0.0554, wR_2 = 0.1464	R_1 = 0.0575, wR_2 = 0.1480
Final R indexes [all data]	R_1 = 0.0798, wR_2 = 0.1718	R_1 = 0.0821, wR_2 = 0.1707
Largest diff. peak/hole / e Å^{-3}	0.15/-0.19	0.18/-0.14
Flack parameter	-0.04(16)	-0.2(3)
CCDC No.	2377057	2377056

Table S2 Bond Lengths for *N*-lauroyl-(*S*)-alanine (*S*)- and (*R*)- nornicotine salts.

***N*-lauroyl-(*S)*-alanine (*S*)-nornicotine**			***N*-lauroyl-(*S)*-alanine (*R*)-nornicotine**		
O1	C1	1.259(4)	O1	C1	1.272(5)
O2	C1	1.225(4)	O2	C1	1.225(5)
O3	C4	1.227(4)	O3	C4	1.225(4)
N1	C19	1.503(4)	N1	C19	1.497(6)
N1	C16	1.487(5)	N1	C16	1.501(6)
N2	C21	1.331(7)	N2	C21	1.334(7)
N2	C22	1.318(8)	N2	C22	1.294(10)
N3	C2	1.444(5)	N3	C2	1.455(4)
N3	C4	1.344(5)	N3	C4	1.337(5)
C1	C2	1.519(4)	C1	C2	1.514(5)
C2	C3	1.510(6)	C2	C3	1.525(5)
C4	C5	1.513(5)	C4	C5	1.506(5)
C5	C6	1.487(5)	C5	C6	1.493(5)
C6	C7	1.516(6)	C6	C7	1.504(5)
C7	C8	1.505(6)	C7	C8	1.497(5)
C8	C9	1.506(7)	C8	C9	1.516(5)
C9	C10	1.510(6)	C9	C10	1.496(5)
C10	C11	1.505(7)	C10	C11	1.503(5)
C11	C12	1.495(7)	C11	C12	1.485(6)
C12	C13	1.518(8)	C12	C13	1.522(6)
C13	C14	1.444(8)	C13	C14	1.450(7)
C14	C15	1.498(9)	C14	C15	1.521(7)
C16	C17	1.525(6)	C16	C17	1.483(7)
C17	C18	1.519(6)	C17	C18	1.491(8)
C18	C19	1.519(5)	C18	C19	1.496(7)
C19	C20	1.492(5)	C19	C20	1.503(7)
C20	C21	1.389(5)	C20	C21	1.355(7)
C20	C24	1.387(6)	C20	C24	1.359(8)
C22	C23	1.383(8)	C24	C23	1.393(11)
C23	C24	1.364(7)	C23	C22	1.348(12)

Table S3 Bond Angles for *N*-lauroyl-*S*-alanine (*S*)- and (*R*)- nornicotine salts.

***N*-lauroyl-*S*-alanine (*R*)-nornicotine**

O1	C1	O2	122.1(3)
O1	C1	C2	118.3(2)
O2	C1	C2	119.3(3)
O3	C4	N3	122.1(4)
O3	C4	C5	121.9(3)
N1	C16	C17	105.3(3)
N1	C19	C18	101.9(3)
N1	C19	C20	112.5(3)
N2	C21	C20	125.6(5)
N2	C22	C23	124.5(5)
N3	C2	C1	109.6(3)
N3	C2	C3	112.4(3)
N3	C4	C5	115.8(3)
C1	C2	C3	109.3(3)
C2	N3	C4	122.2(3)
C4	C5	C6	114.3(3)
C5	C6	C7	113.8(4)
C6	C7	C8	115.0(4)
C7	C8	C9	114.9(4)
C8	C9	C10	115.1(4)
C9	C10	C11	114.6(5)
C10	C11	C12	116.1(5)
C11	C12	C13	114.6(5)
C12	C13	C14	117.2(6)
C13	C14	C15	114.0(7)
C16	C17	C18	106.0(3)
C16	N1	C19	105.6(3)
C17	C18	C19	105.5(3)
C19	C20	C21	120.2(4)
C19	C20	C24	123.2(3)
C20	C19	C18	117.8(3)
C20	C24	C23	119.3(4)
C21	C20	C24	116.5(4)
C21	N2	C22	115.4(5)
C22	C23	C24	118.7(5)

***N*-lauroyl-*S*-alanine (*S*)-nornicotine**

O1	C1	O2	122.8(4)
O1	C1	C2	116.4(4)
O2	C1	C2	120.8(3)
O3	C4	N3	122.6(4)
O3	C4	C5	122.5(4)
N1	C16	C17	106.4(4)
N1	C19	C18	103.0(4)
N1	C19	C20	114.0(4)
N2	C21	C20	125.5(6)
N2	C22	C23	123.7(8)
N3	C2	C1	110.1(3)
N3	C2	C3	111.6(3)
N3	C4	C5	114.8(3)
C1	C2	C3	109.7(4)
C2	N3	C4	122.8(3)
C4	C5	C6	115.8(3)
C5	C6	C7	113.5(3)
C6	C7	C8	116.1(4)
C7	C8	C9	114.8(4)
C8	C9	C10	116.0(4)
C9	C10	C11	114.9(4)
C10	C11	C12	116.5(4)
C11	C12	C13	115.0(5)
C12	C13	C14	115.0(5)
C13	C14	C15	114.2(6)
C16	C17	C18	106.8(4)
C16	N1	C19	105.1(4)
C17	C18	C19	106.6(5)
C19	C20	C21	125.6(5)
C19	C20	C24	117.3(6)
C20	C19	C18	117.1(5)
C20	C24	C23	118.2(8)
C21	C20	C24	117.1(6)
C21	N2	C22	116.2(7)
C22	C23	C24	119.3(7)

Table S4 Torsion Angles for *N*-lauroyl-*S*-alanine (*S*)- and (*R*)- nornicotine salts.

***N*-lauroyl-*S*-alanine (*S*)-nornicotine**					***N*-lauroyl-*S*-alanine (*R*)-nornicotine**				
O1	C1	C2	N3	159.2(3)	O1	C1	C2	N3	171.2(3)
O1	C1	C2	C3	-77.1(4)	O1	C1	C2	C3	-65.6(5)
O2	C1	C2	N3	-26.2(5)	O2	C1	C2	N3	-10.8(5)
O2	C1	C2	C3	97.5(4)	O2	C1	C2	C3	112.4(5)
O3	C4	C5	C6	-31.5(6)	O3	C4	C5	C6	-26.3(7)
N1	C16	C17	C18	8.2(4)	N1	C16	C17	C18	-6.4(7)
N1	C19	C18	C17	-34.4(4)	N1	C19	C18	C17	31.0(6)
N1	C19	C20	C21	117.9(3)	N1	C19	C20	C21	38.0(7)
N1	C19	C20	C24	-64.2(4)	N1	C19	C20	C24	-145.0(6)
N2	C21	C20	C24	0.5(6)	N2	C21	C20	C24	2.5(10)
N2	C21	C20	C19	178.4(4)	N2	C21	C20	C19	179.5(6)
N2	C22	C23	C24	-0.6(9)	N2	C22	C23	C24	0.5(16)
N3	C4	C5	C6	152.7(4)	N3	C4	C5	C6	157.6(4)
C2	N3	C4	O3	0.4(6)	C2	N3	C4	O3	-4.3(7)
C2	N3	C4	C5	176.2(3)	C2	N3	C4	C5	171.8(4)
C4	N3	C2	C1	-158.2(3)	C4	N3	C2	C1	-141.7(4)
C4	N3	C2	C3	80.0(5)	C4	N3	C2	C3	96.2(5)
C4	C5	C6	C7	174.7(4)	C4	C5	C6	C7	172.3(4)
C5	C6	C7	C8	-179.4(4)	C5	C6	C7	C8	-179.9(4)
C6	C7	C8	C9	177.2(4)	C6	C7	C8	C9	176.2(4)
C7	C8	C9	C10	179.4(5)	C7	C8	C9	C10	178.5(4)
C8	C9	C10	C11	178.3(5)	C8	C9	C10	C11	177.6(4)
C9	C10	C11	C12	-177.7(5)	C9	C10	C11	C12	-179.6(4)
C10	C11	C12	C13	174.5(5)	C10	C11	C12	C13	177.0(4)
C11	C12	C13	C14	-175.5(6)	C11	C12	C13	C14	-178.0(5)
C12	C13	C14	C15	175.0(6)	C12	C13	C14	C15	178.8(5)
C16	N1	C19	C18	40.2(3)	C16	N1	C19	C20	-162.6(4)
C16	N1	C19	C20	167.3(3)	C16	N1	C19	C18	-34.7(5)
C16	C17	C18	C19	16.5(4)	C16	C17	C18	C19	-15.5(8)
C18	C19	C20	C24	53.8(5)	C18	C19	C20	C24	94.7(7)
C18	C19	C20	C21	-124.0(4)	C18	C19	C20	C21	-82.2(6)
C19	N1	C16	C17	-30.3(4)	C19	N1	C16	C17	25.8(6)
C19	C20	C24	C23	-177.9(4)	C19	C20	C24	C23	-178.9(7)
C20	C19	C18	C17	-158.0(3)	C20	C19	C18	C17	156.9(5)
C20	C24	C23	C22	0.1(7)	C20	C24	C23	C22	0.3(14)
C21	N2	C22	C23	1.0(9)	C21	N2	C22	C23	0.2(13)
C21	C20	C24	C23	0.0(6)	C21	C20	C24	C23	-1.7(11)
C22	N2	C21	C20	-0.9(8)	C22	N2	C21	C20	-1.8(11)

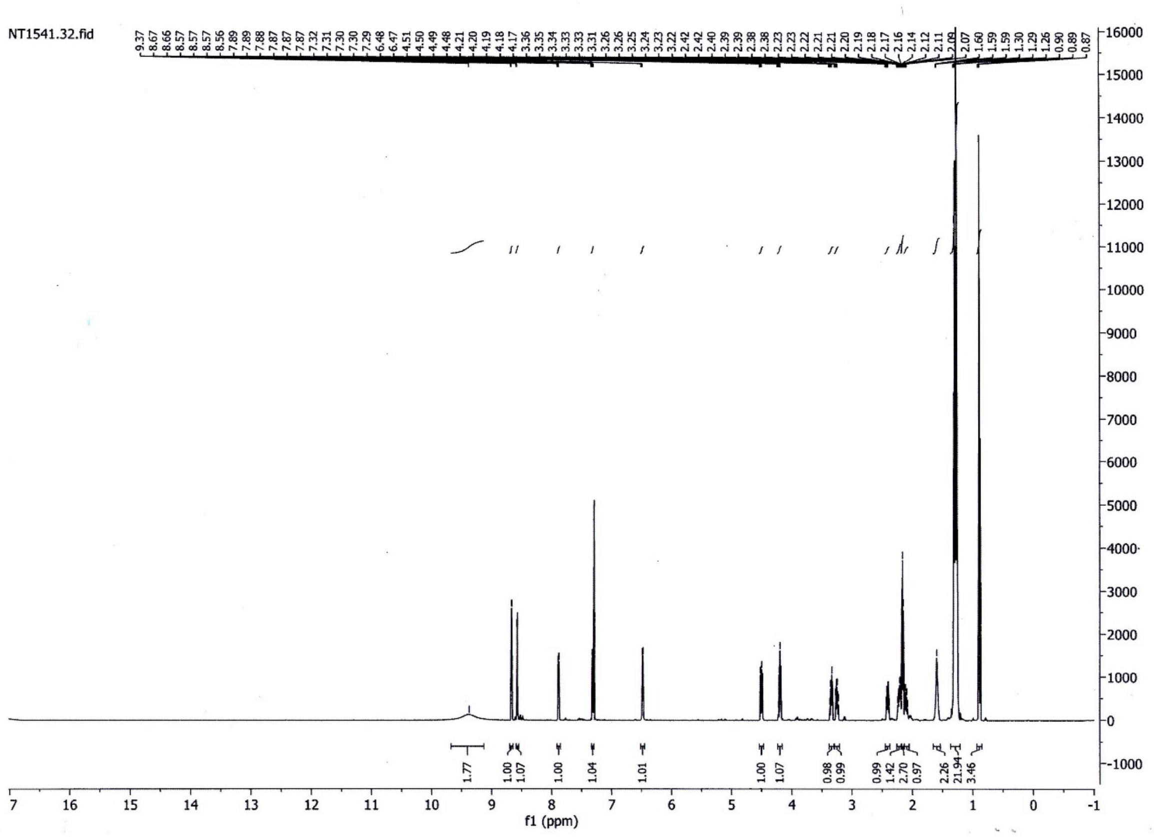

Figure S3. ^{1}H NMR spectrum of *N*-lauroyl-*(S)*-alanine•*(R)*-nornicotine salt

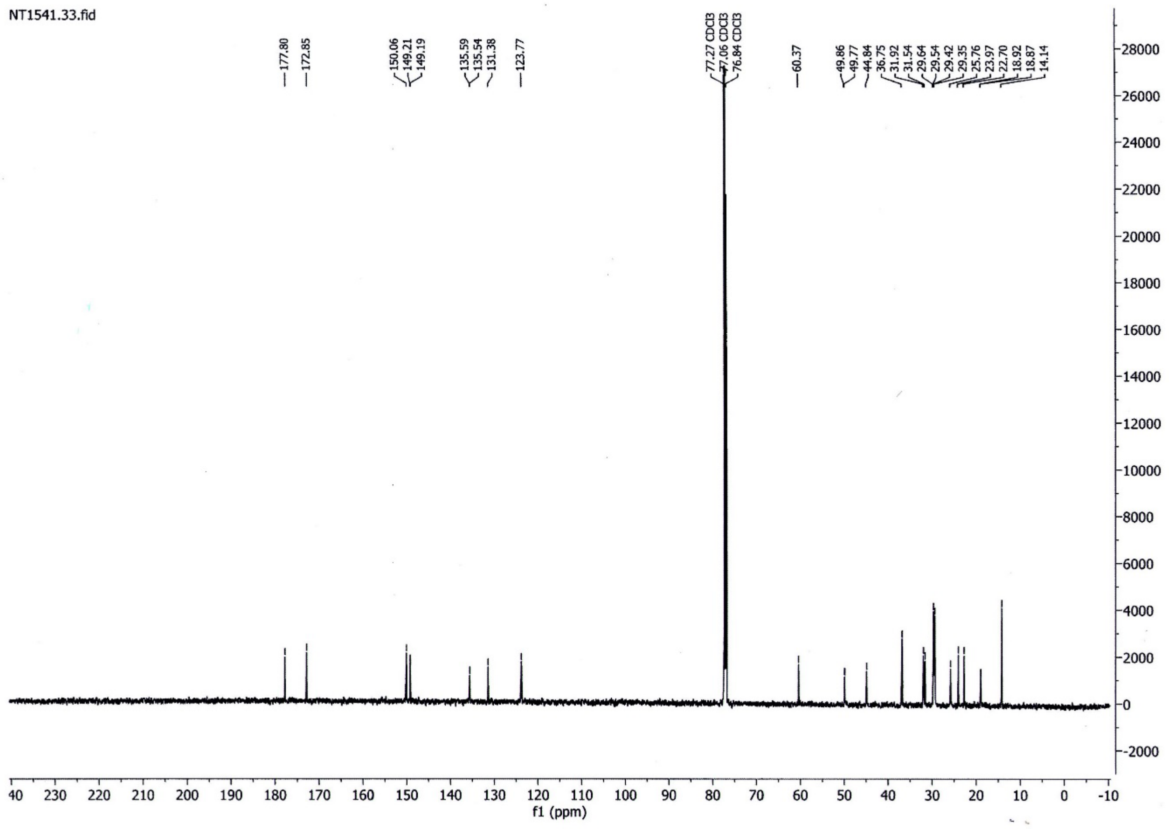

Figure S4. ^{13}C NMR spectrum of *N*-lauroyl-*(S)*-alanine•*(R)*-nornicotine salt

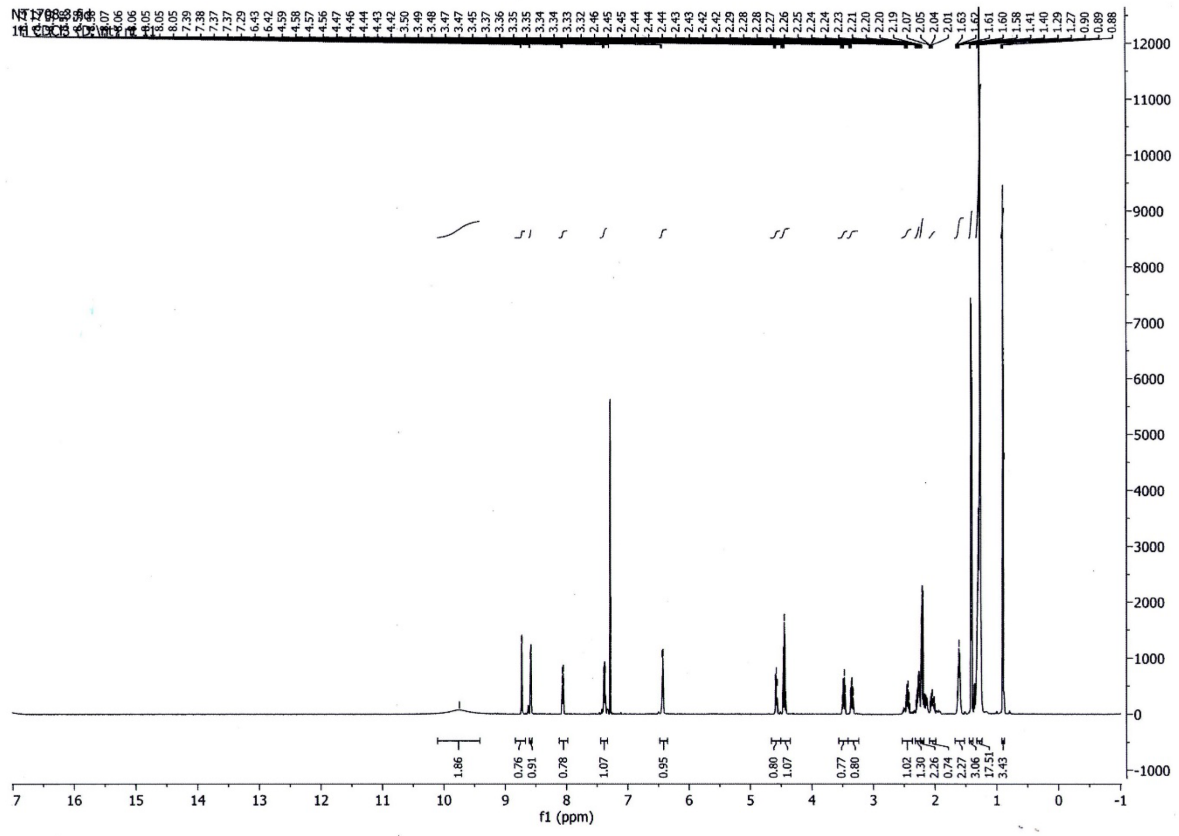

Figure S5. ^{1}H NMR spectrum of *N*-lauroyl-*(R)*-alanine•*(S)*-nornicotine salt

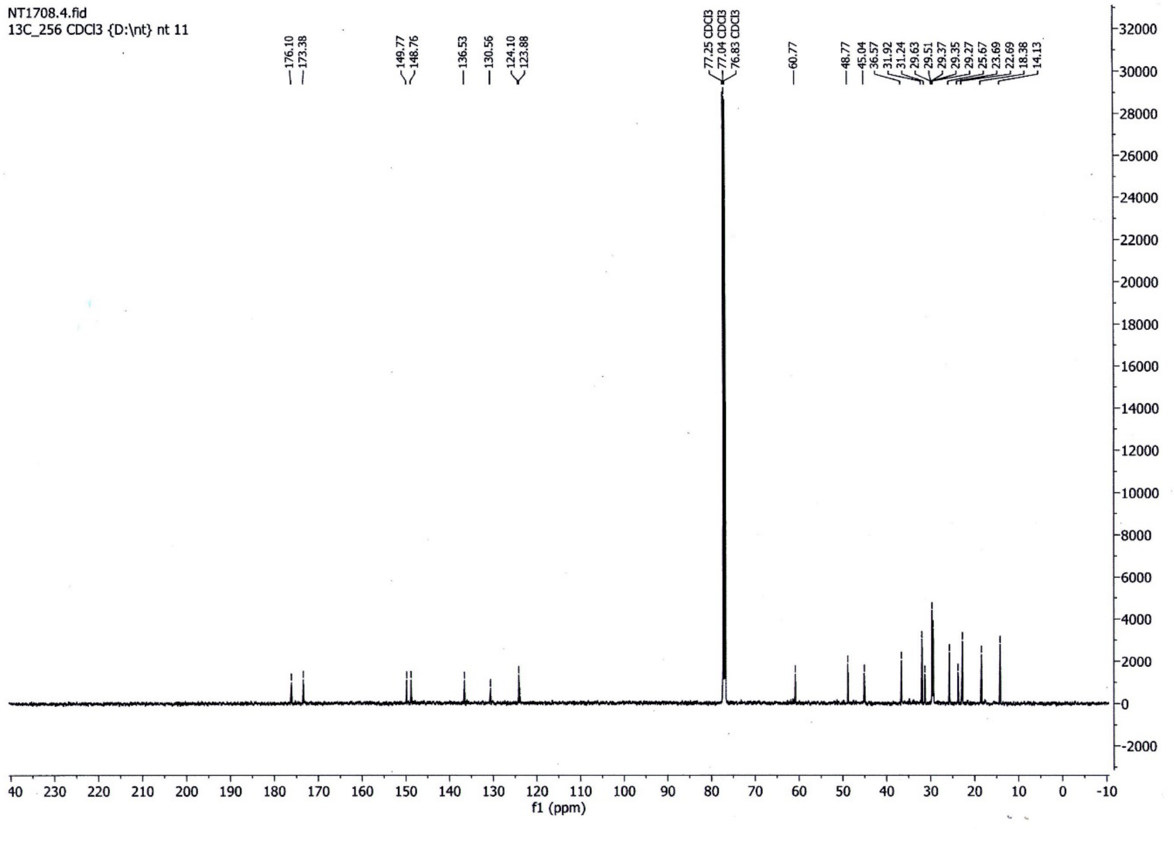

Figure S6. ^{13}C NMR spectrum of *N*-lauroyl-*(R)*-alanine•*(S)*-nornicotine salt

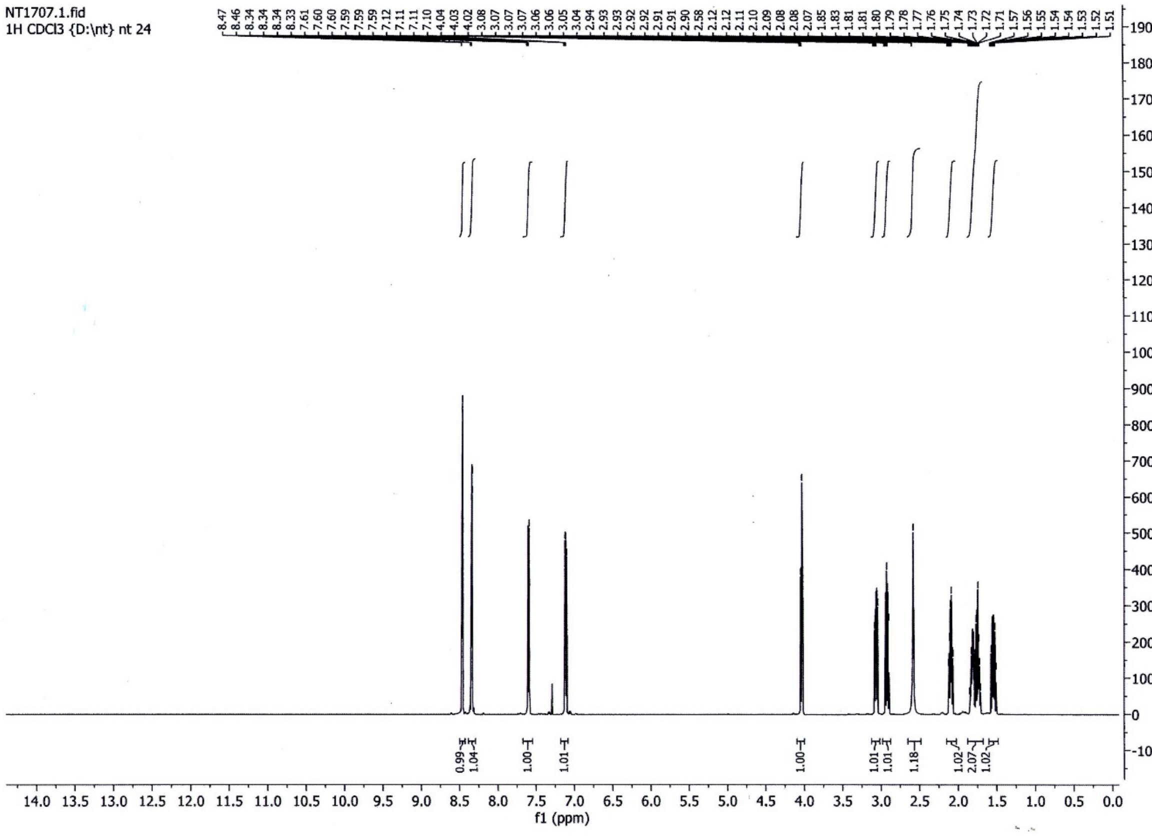

Figure S7. ^{1}H NMR spectrum of (*R*)-nornicotine

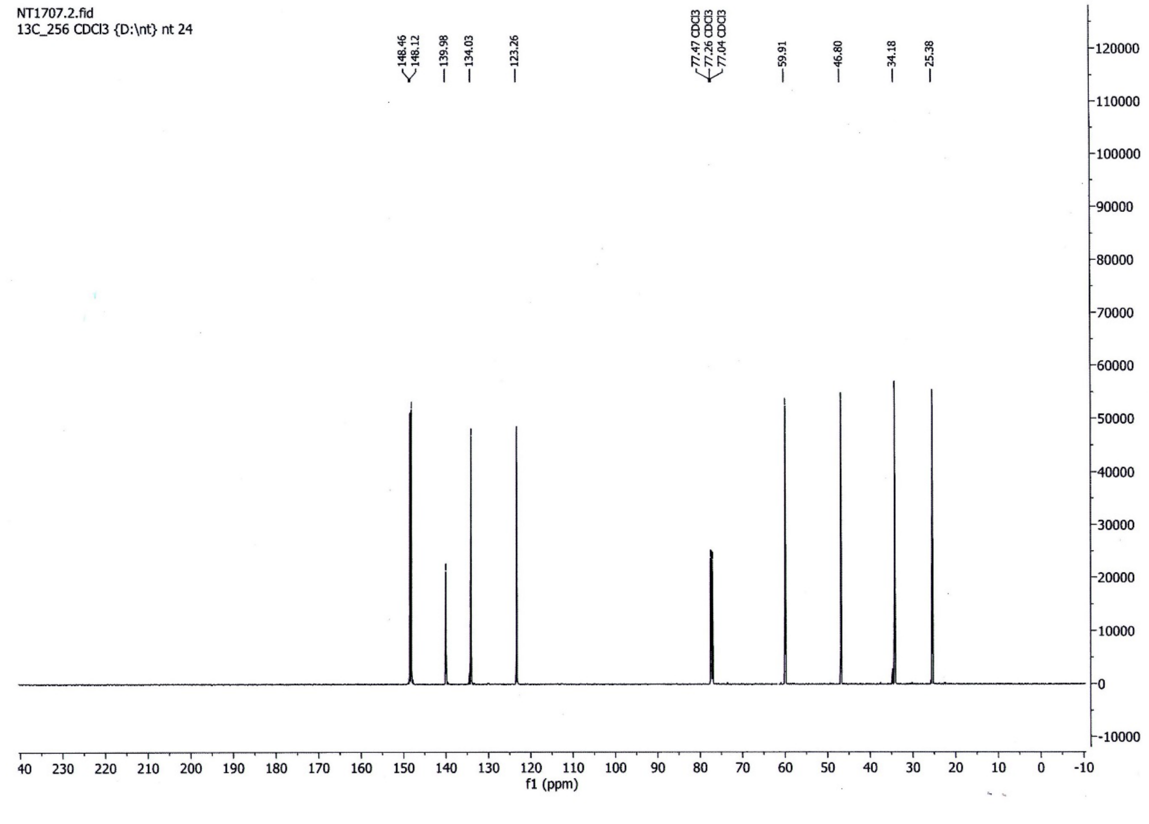

Figure S8. ^{13}C NMR spectrum of (*R*)-nornicotine

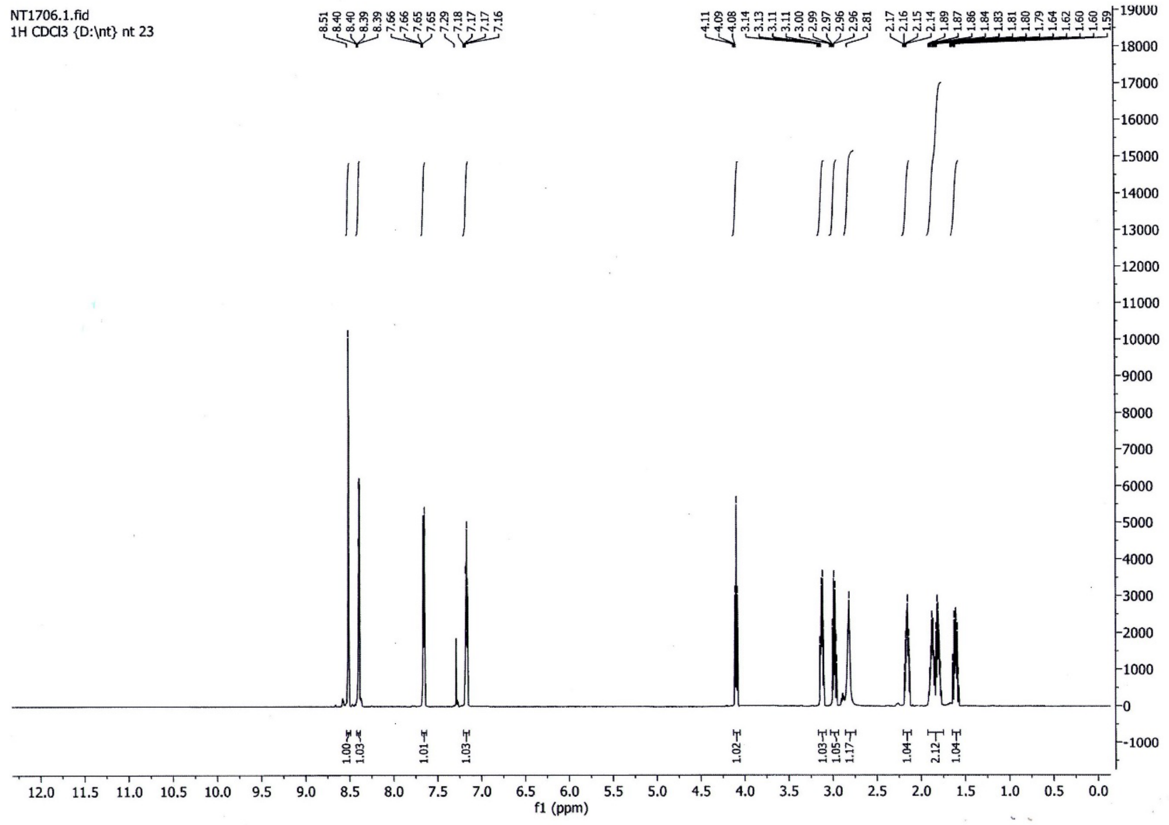

Figure S9. ^{1}H NMR spectrum of (*S*)-nornicotine

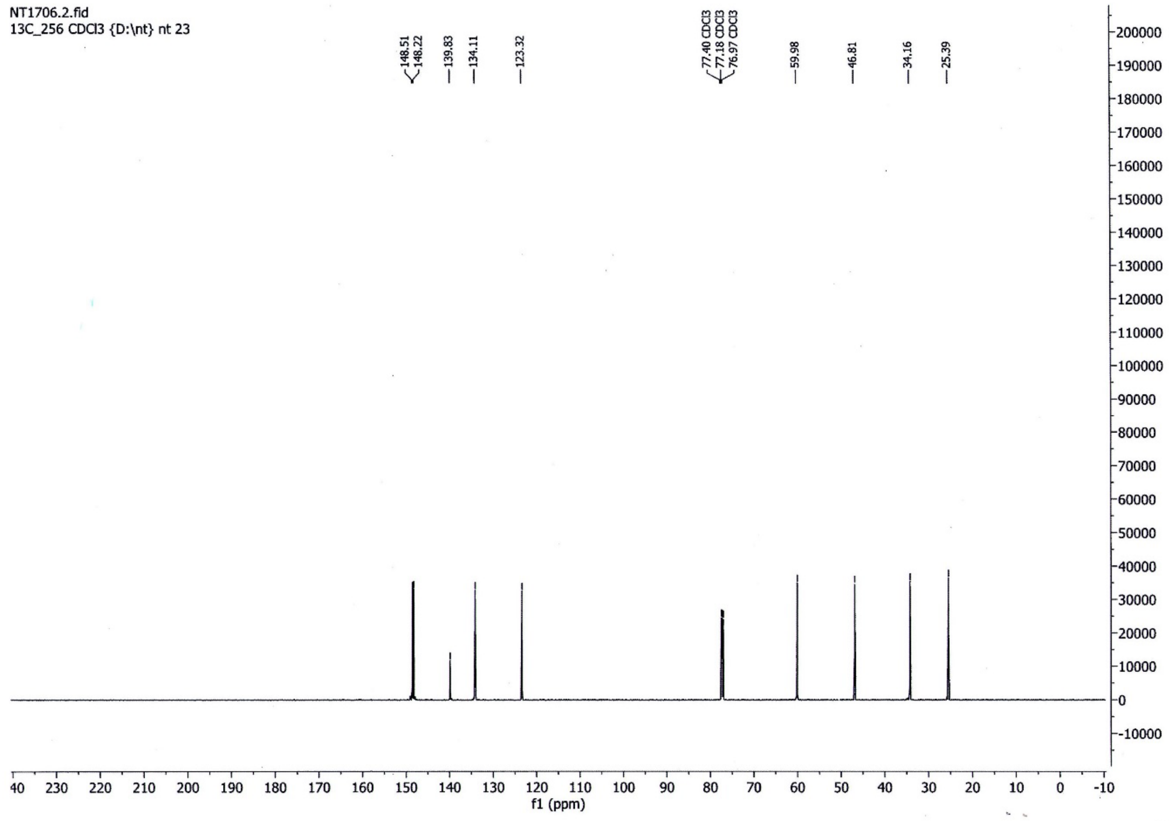

Figure S10. ^{13}C NMR spectrum of (*S*)-nornicotine

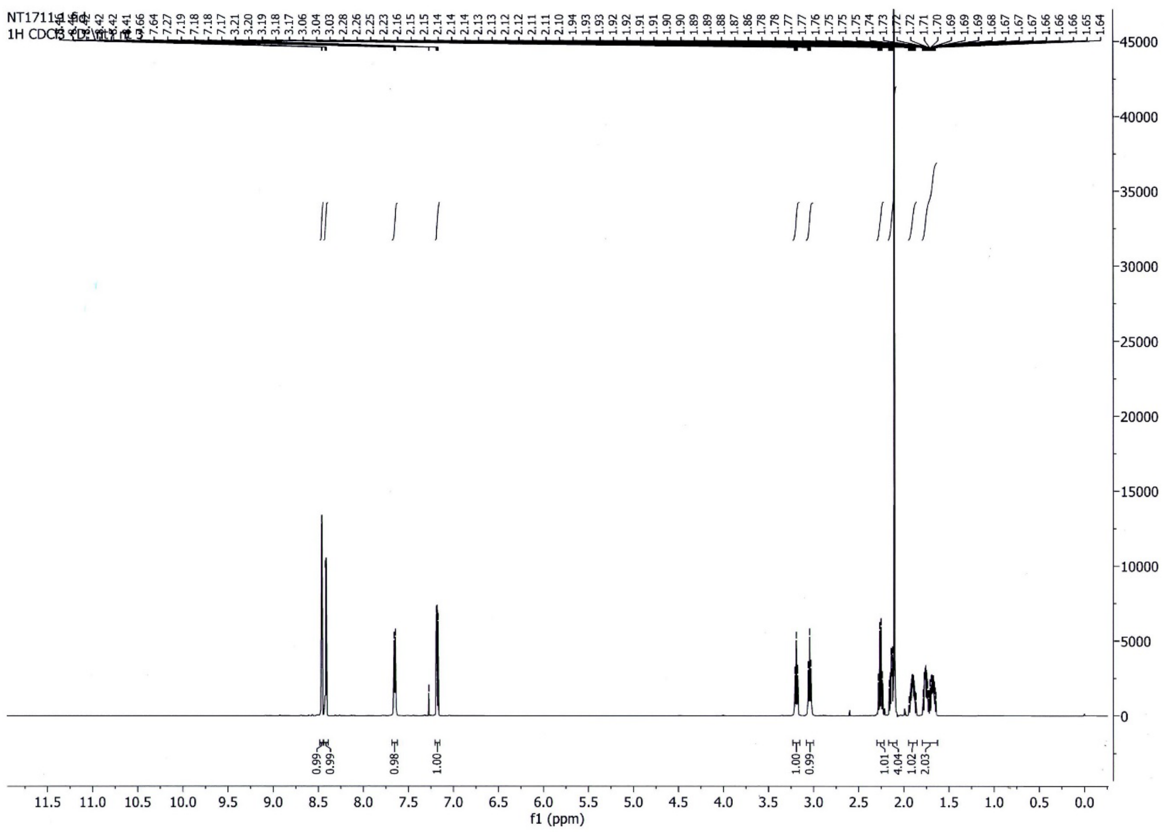

Figure S11. ^{1}H NMR spectrum of (*S*)-nicotine

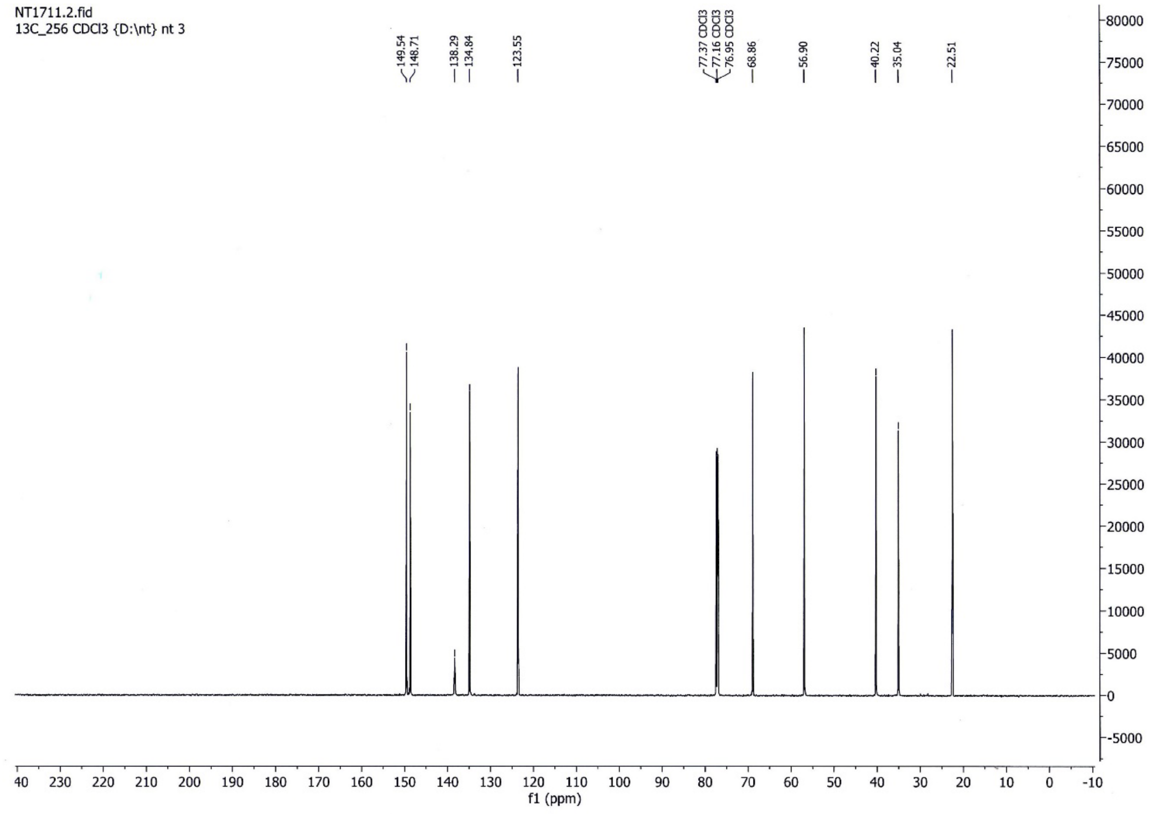

Figure S12. ^{13}C NMR spectrum of (*S*)-nicotine

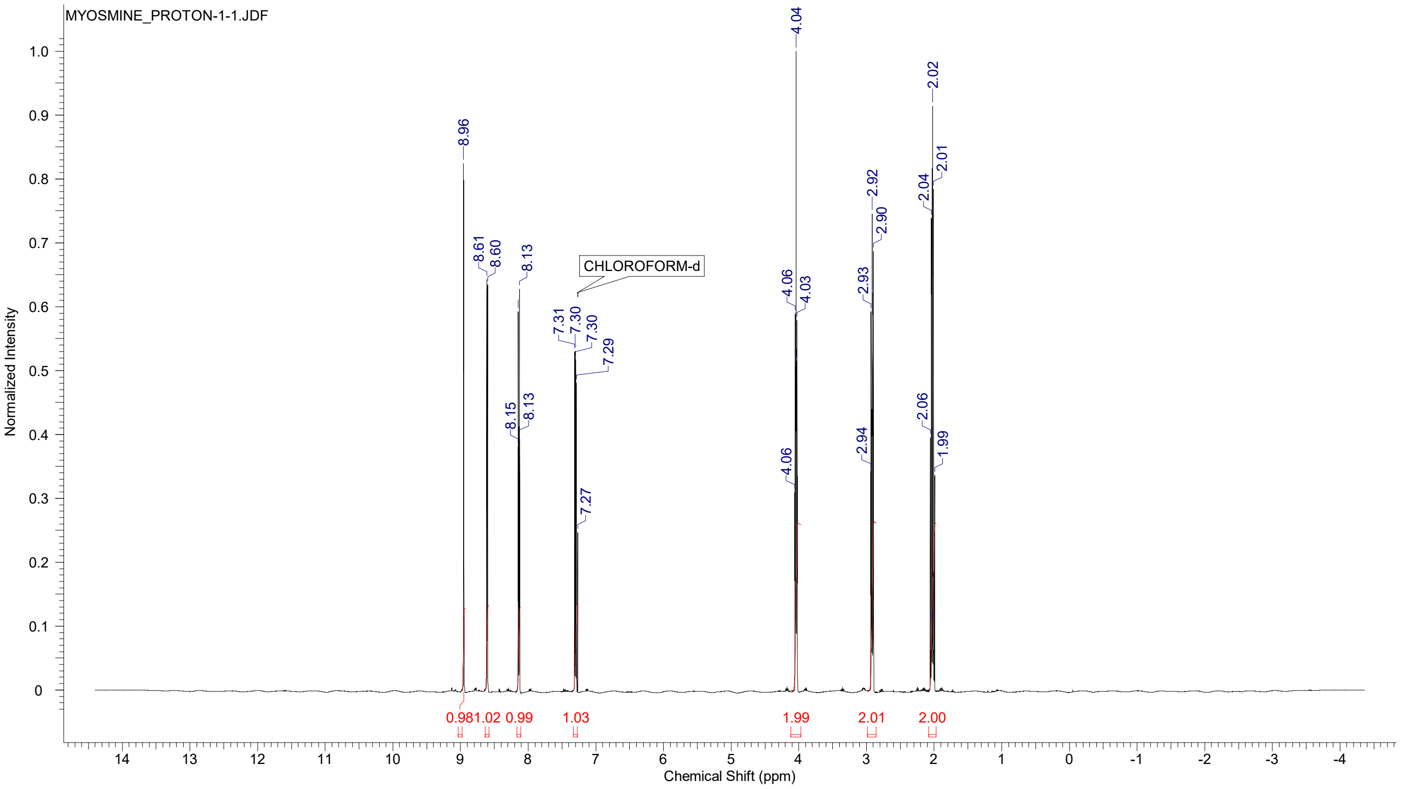

Figure S13. ^{1}H NMR spectrum of myosmine

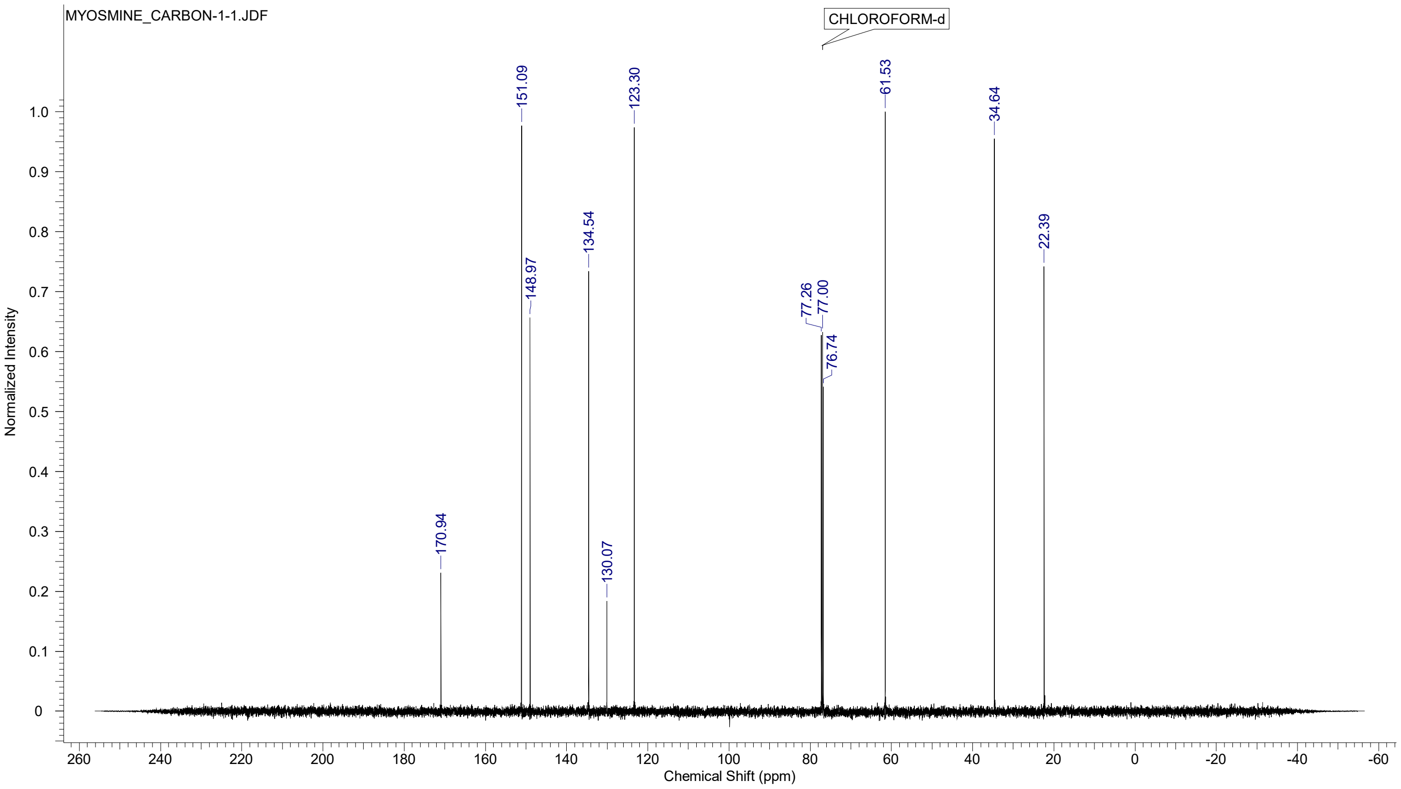

Figure S14. ^{13}C NMR spectrum of myosmine

Formula Predictor Report

Printed at 08.11.2024 12:12:43

Formula Predictor Result	**C9 H10 N2**
Mass	147.09105
Error Margin	30 ppm
DBE Range	Not Used
Electron Ions	Both configurations
HC Ratio	Not Used
Nitrogen Rule	Not Used

#	Score	Pred. (M)	Pred. m/z	Meas. m/z	Diff. (mDa)	Formulae (M)	Ion	Diff. (ppm)	Iso Score	DBE
1	97.37	146.08440	147.09167	147.09105	-0.62	C9 H10 N2	[M+H]+	-4.248	97.08	6.0

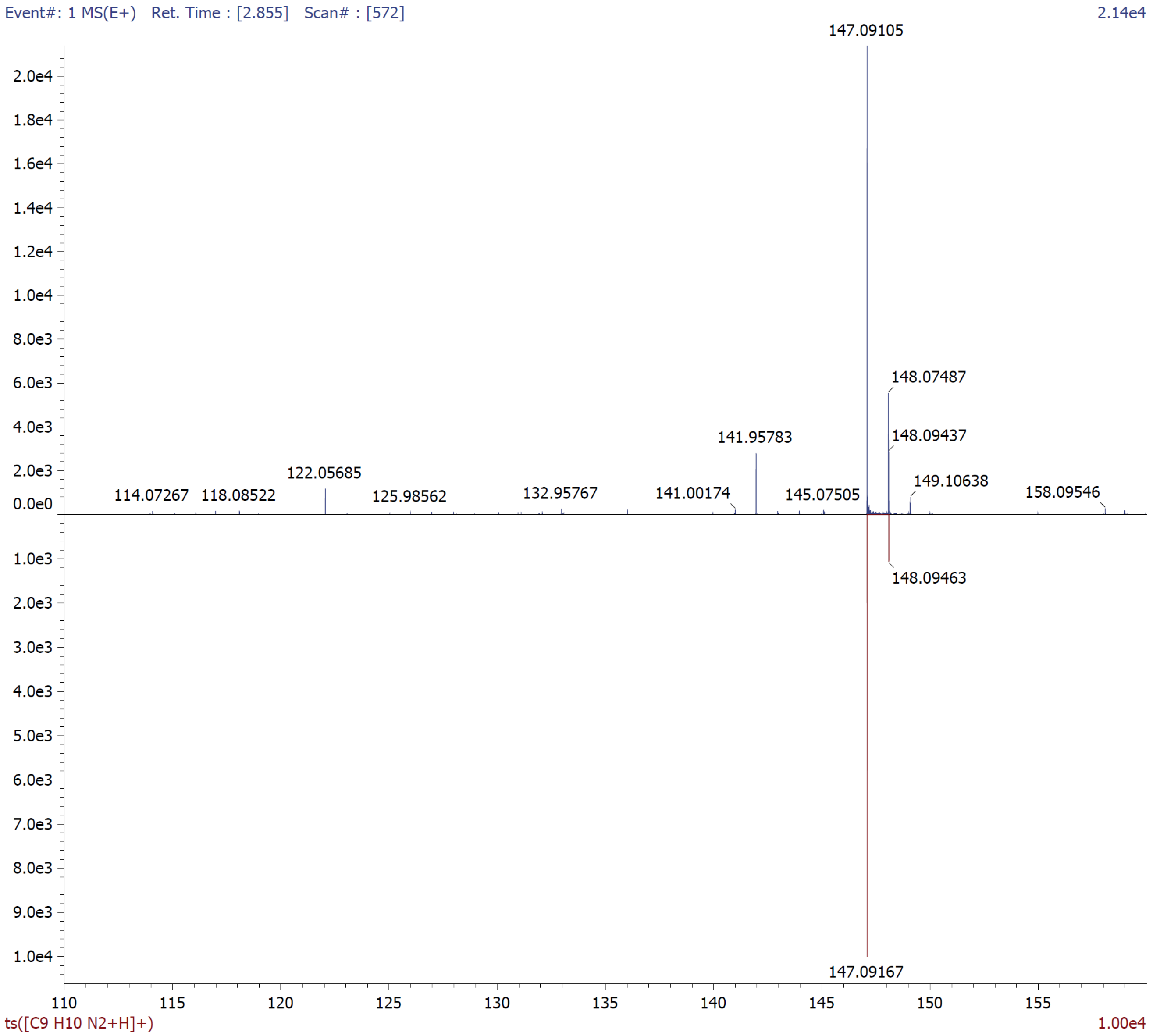

Page 1 of 1

Figure S15. HRMS analysis of myosmine (Q-Tof)

Formula Predictor Report

Printed at 08.11.2024 12:14:08

Formula Predictor Result	**C9 H12 N2**
Mass	149.10677
Error Margin	30 ppm
DBE Range	Not Used
Electron Ions	Both configurations
HC Ratio	Not Used
Nitrogen Rule	Not Used

#	Score	Pred. (M)	Pred. m/z	Meas. m/z	Diff. (mDa)	Formulae (M)	Ion	Diff. (ppm)	Iso Score	DBE
1	88.95	148.10005	149.10732	149.10677	-0.55	C9 H12 N2	[M+H]+	-3.721	87.72	5.0

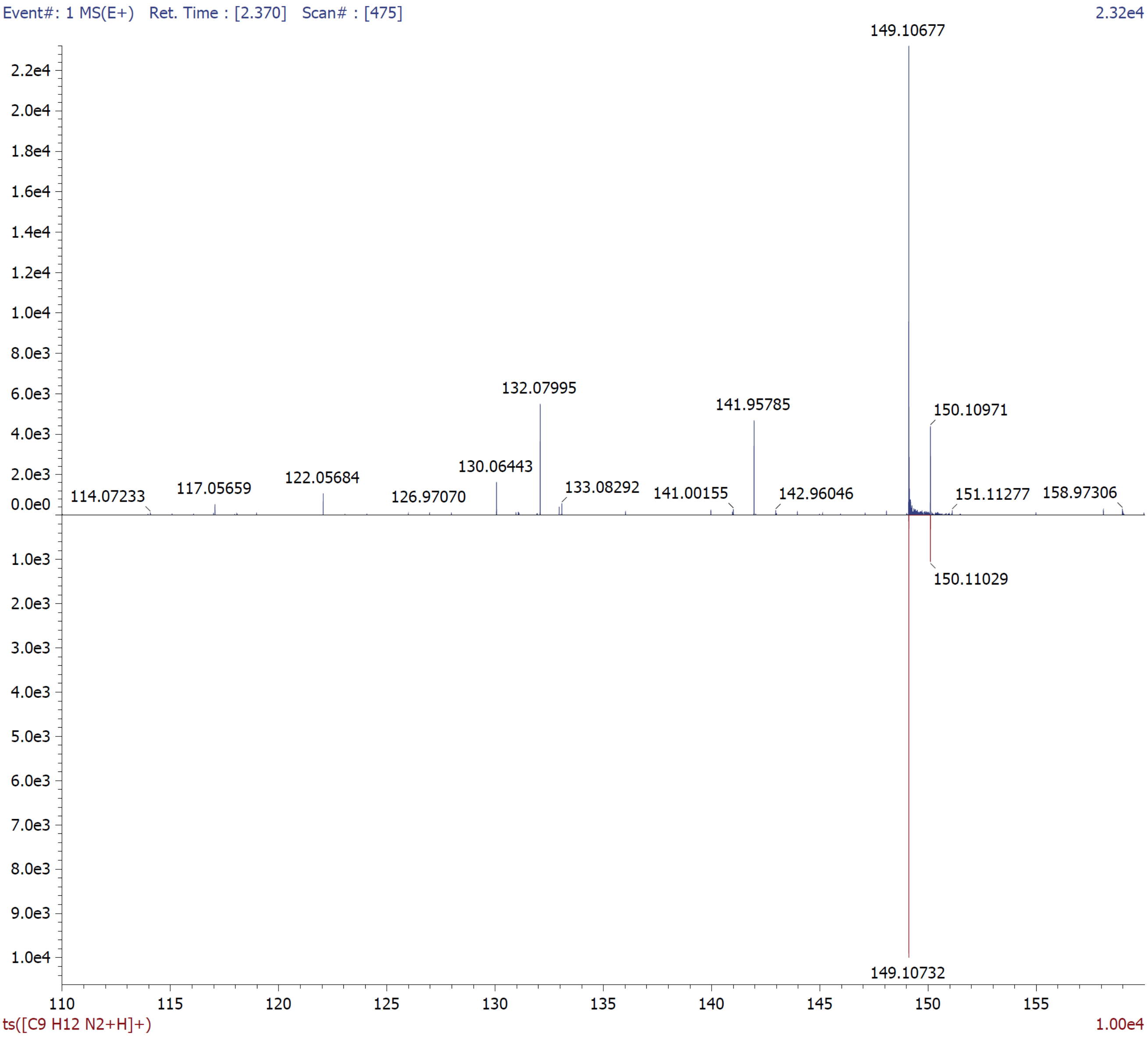

Page 1 of 1

Figure S16. HRMS analysis of nornicotine (Q-Tof)

Formula Predictor Report

Printed at 08.11.2024 12:37:24

Formula Predictor Result	**C10 H14 N2**
Mass	163.12247
Error Margin	30 ppm
DBE Range	Not Used
Electron Ions	Both configurations
HC Ratio	Not Used
Nitrogen Rule	Not Used

#	Score	Pred. (M)	Pred. m/z	Meas. m/z	Diff. (mDa)	Formulae (M)	Ion	Diff. (ppm)	Iso Score	DBE
1	98.16	162.11570	163.12297	163.12247	-0.50	C10 H14 N2	[M+H]+	-3.095	97.96	5.0

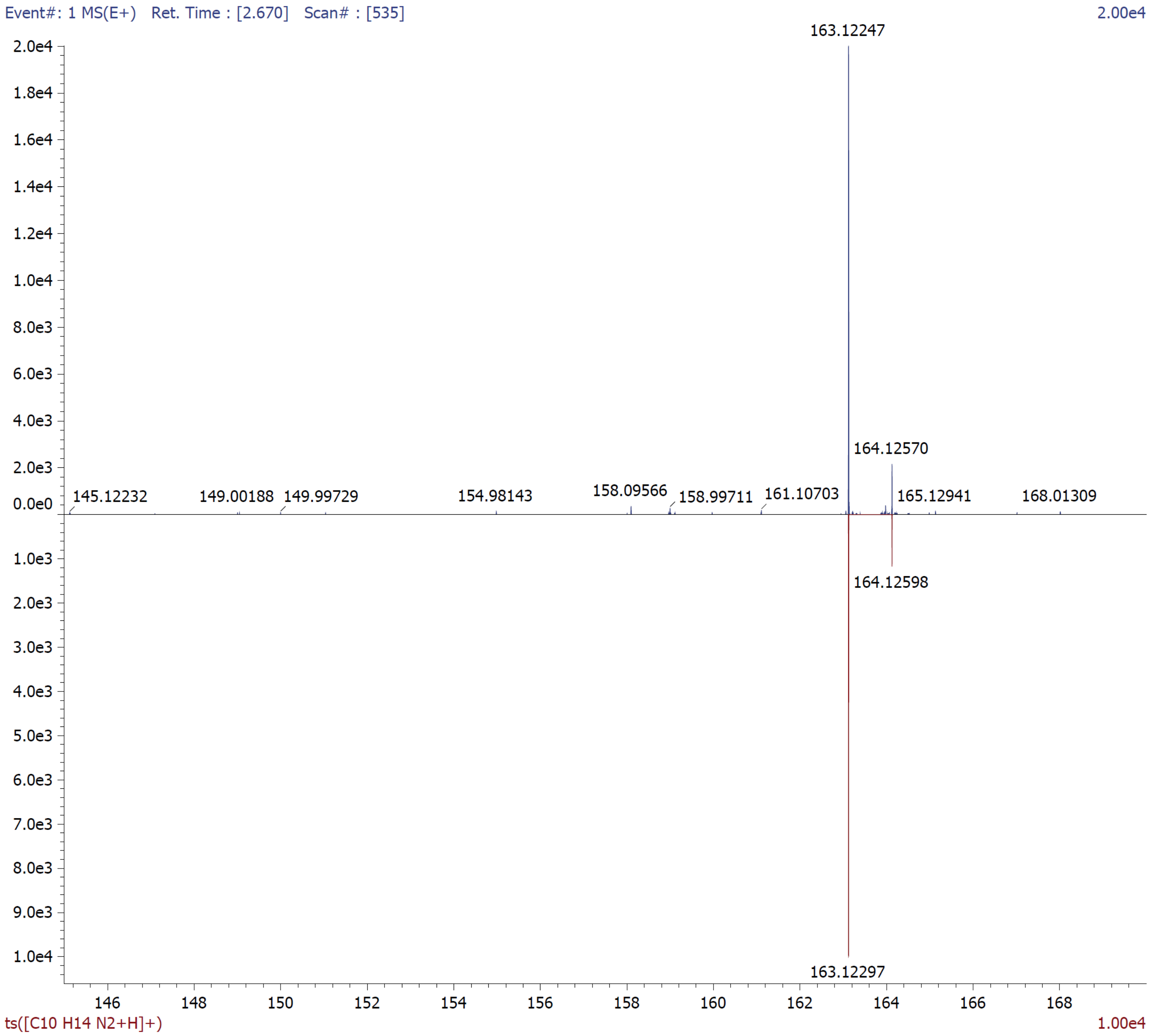

Page 1 of 1

Figure S17. HRMS analysis of nicotine (Q-Tof)

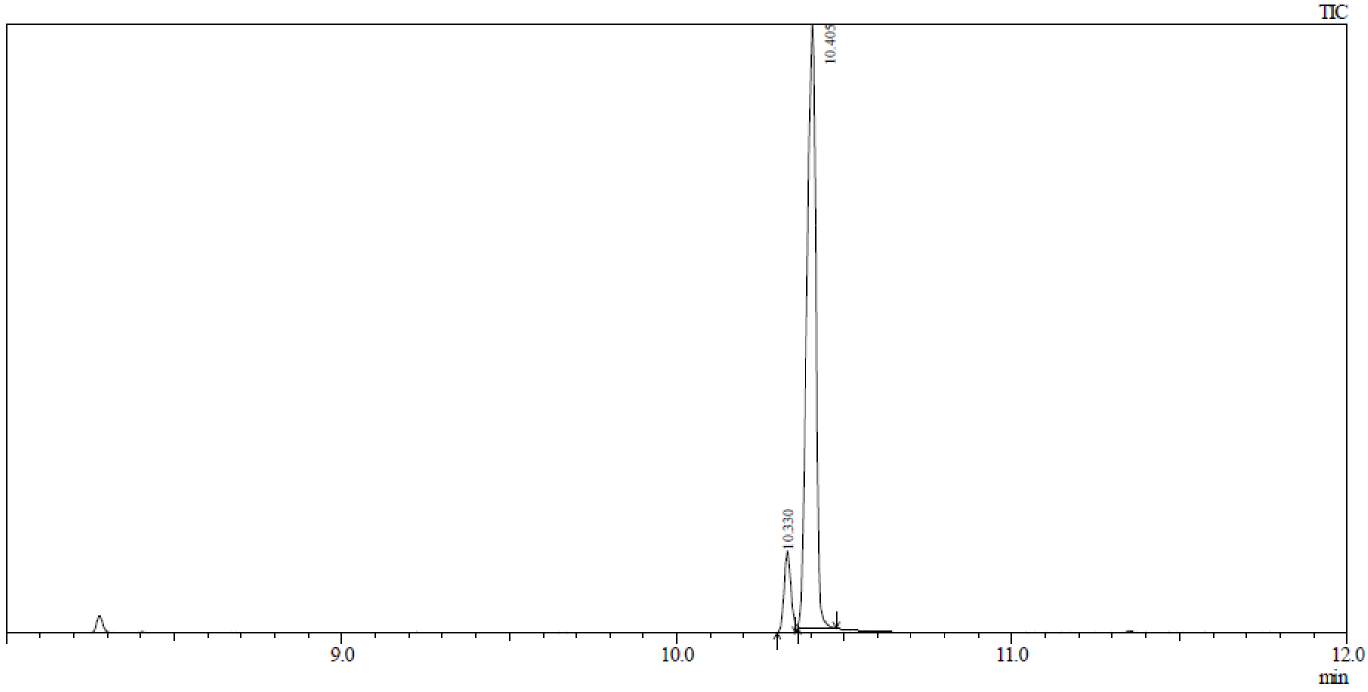

Peak Report TIC

Peak#	R.Time	I.Time	F.Time	Area	Area%	Height	Height%	A/H	Mark	Name
1	10.330	10.300	10.355	2753470	8.95	1971287	11.88	1.40	MI	
2	10.405	10.360	10.475	28018116	91.05	14619001	88.12	1.92	MI	
				30771586	100.00	16590288	100.00			

Spectrum

Line#:1 R.Time:10.330(Scan#:1367)
MassPeaks:150
RawMode:Averaged 10.320-10.340(1365-1369) BasePeak:119(312170)
BG Mode:Averaged 10.435-10.545(1388-1410) Group 1 - Event 1 Scan

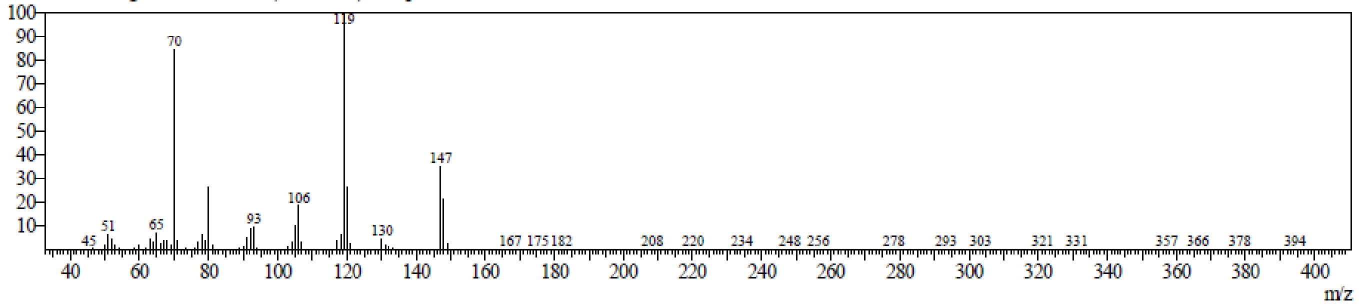

Line#:2 R.Time:10.400(Scan#:1381)
MassPeaks:254
RawMode:Averaged 10.395-10.405(1380-1382) BasePeak:118(3269964)
BG Mode:Averaged 10.725-10.955(1446-1492) Group 1 - Event 1 Scan

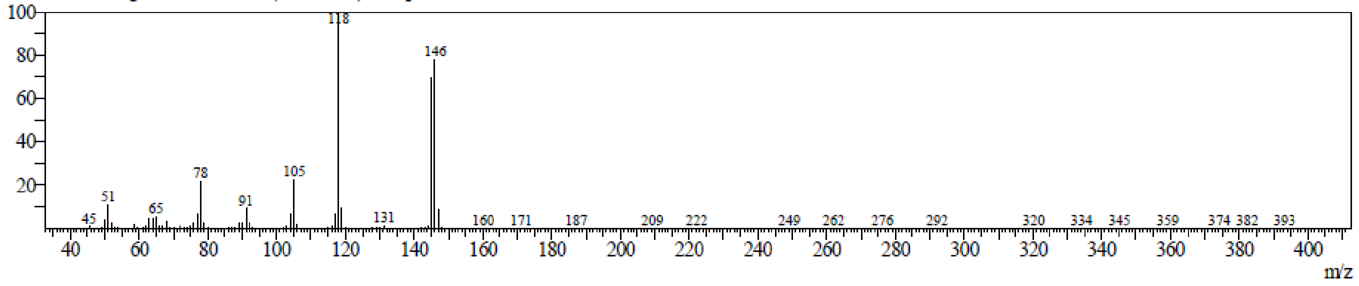

Figure S18. GCMS analysis of reaction mixture of incomplete oxidation of nornicotine to myosmine

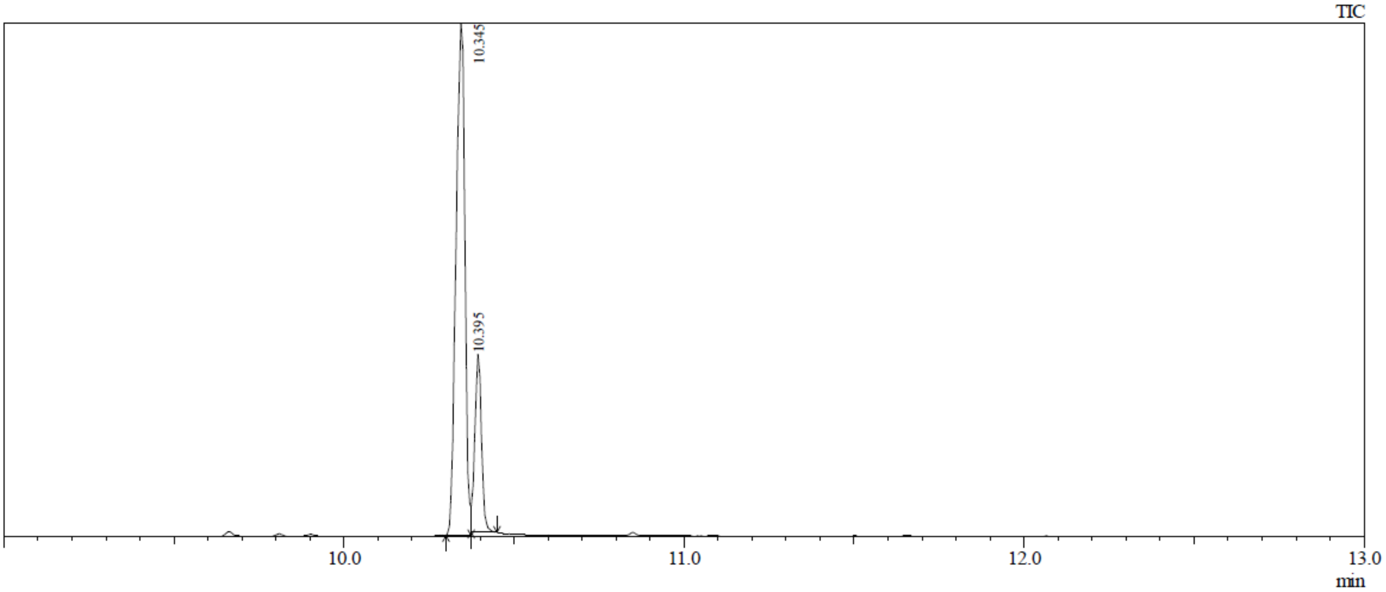

Peak Report TIC

Peak#	R.Time	I.Time	F.Time	Area	Area%	Height	Height%	A/H	Mark	Name
1	10.345	10.300	10.375	26456909	79.58	14494473	74.18	1.83	MI	
2	10.395	10.375	10.450	6788131	20.42	5045527	25.82	1.35	MI	
				33245040	100.00	19540000	100.00			

Spectrum

Line#:1 R.Time:10.345(Scan#:1370)
MassPeaks:280
RawMode:Averaged 10.340-10.350(1369-1371) BasePeak:119(2962176)
BG Mode:Averaged 10.405-10.540(1382-1409) Group 1 - Event 1 Scan

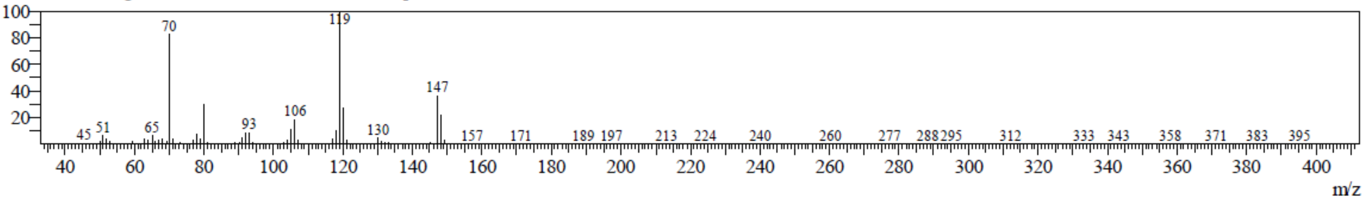

Line#:2 R.Time:10.395(Scan#:1380)
MassPeaks:231
RawMode:Averaged 10.390-10.405(1379-1382) BasePeak:118(942120)
BG Mode:Averaged 10.265-10.320(1354-1365) Group 1 - Event 1 Scan

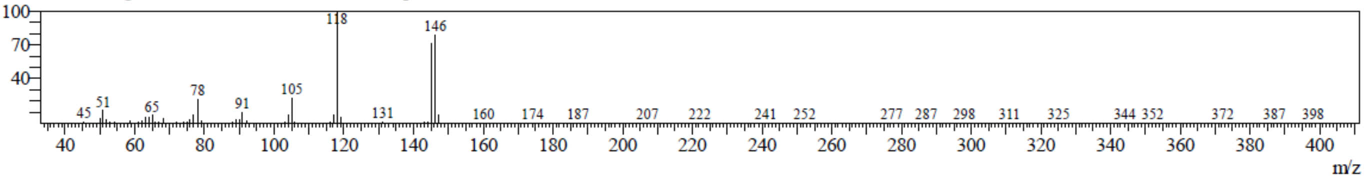

Figure S19. GCMS analysis of reaction mixture of incomplete reduction of myosmine

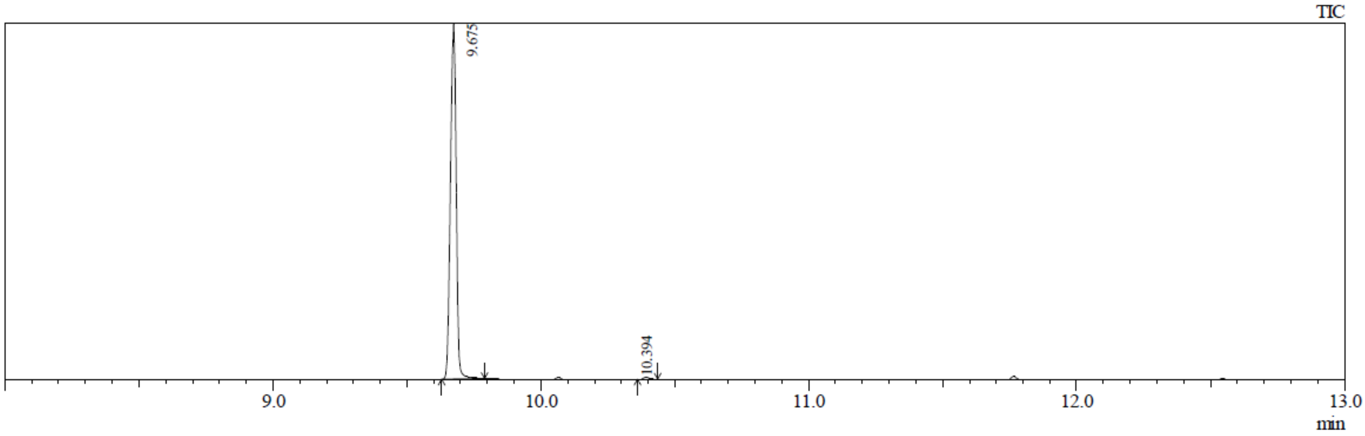

Peak Report TIC

Peak#	R.Time	I.Time	F.Time	Area	Area%	Height	Height%	A/H	Mark	Name
1	9.675	9.630	9.790	25469432	99.48	17000267	99.47	1.50		Pyridine, 3-(1-methyl-2-pyrrolidinyl)-, (S)-
2	10.394	10.360	10.435	134116	0.52	91330	0.53	1.47		Pyridine, 3-(3,4-dihydro-2H-pyrrol-5-yl)-
				25603548	100.00	17091597	100.00			

Spectrum

Line#:1 R.Time:9.675(Scan#:1236)
MassPeaks:232
RawMode:Averaged 9.670-9.680(1235-1237) BasePeak:84(6588287)
BG Mode:Calc. from Peak Group 1 - Event 1 Scan

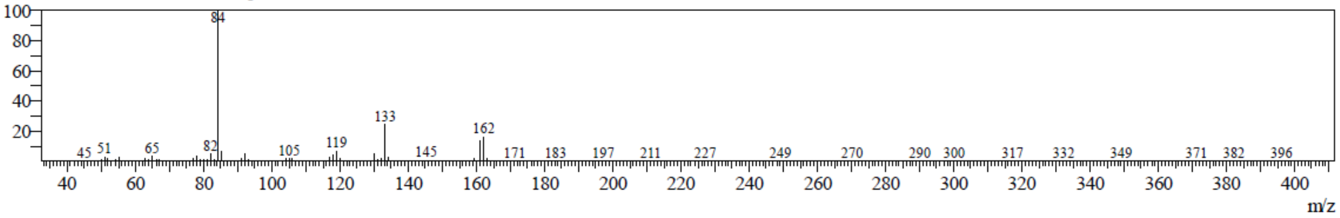

Line#:2 R.Time:10.395(Scan#:1380)
MassPeaks:217
RawMode:Averaged 10.390-10.400(1379-1381) BasePeak:118(20129)
BG Mode:Calc. from Peak Group 1 - Event 1 Scan

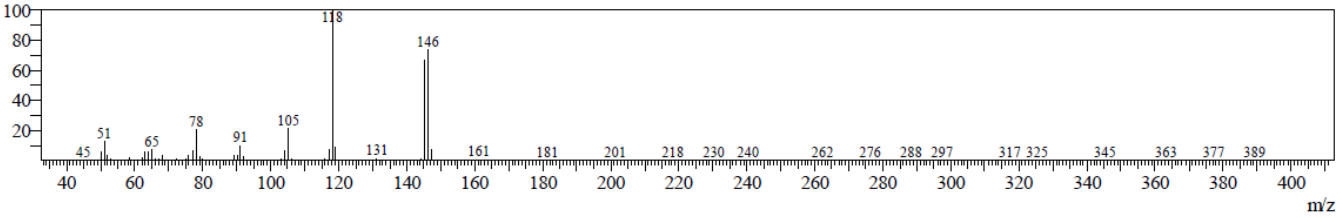

Figure S20. GCMS analysis of nicotine

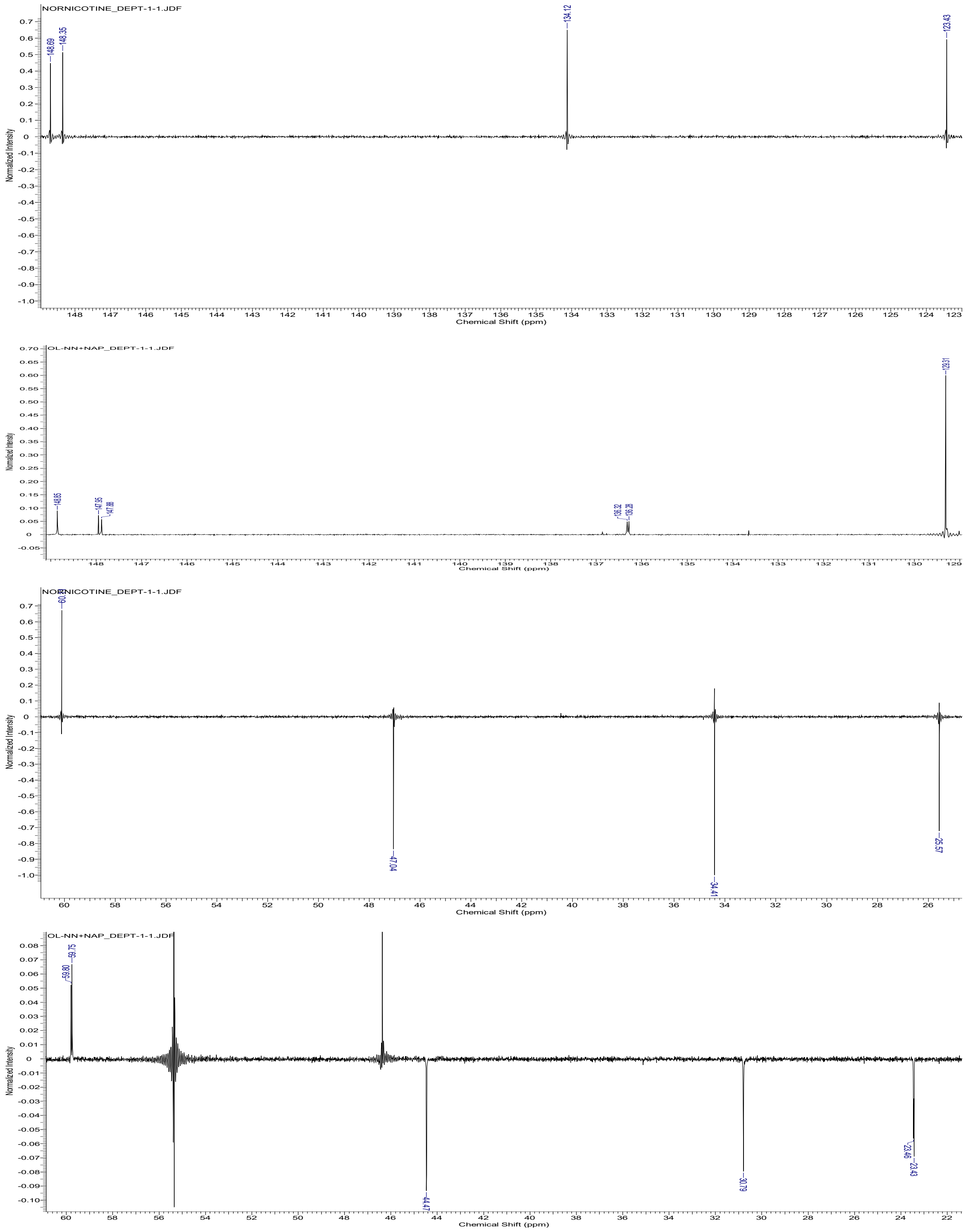

Figure S21. Comparision of charactiristic regions in DEPT135 spectra of rac-nornicotine and *rac*-nornicotine in the presence of naproxen

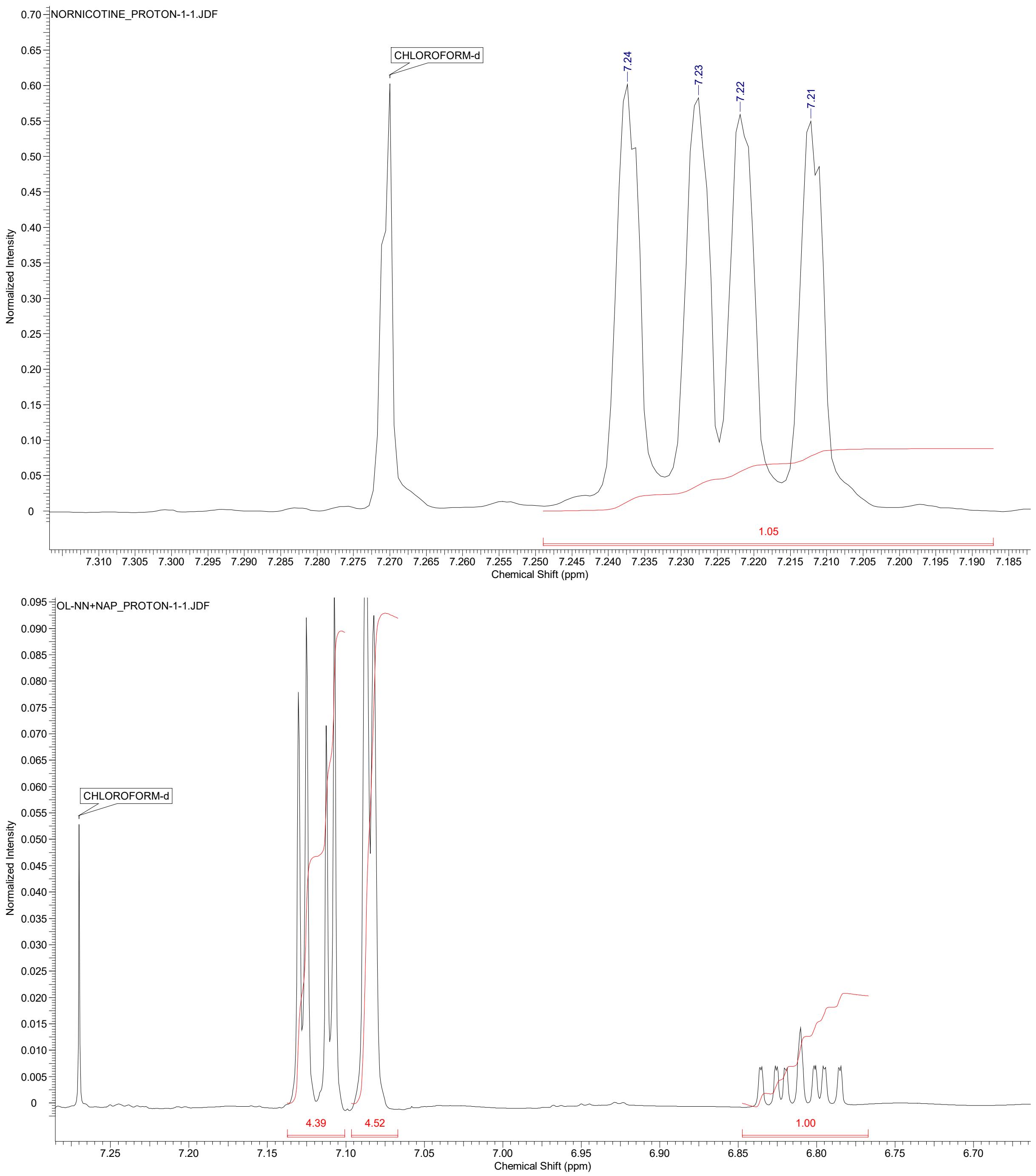

Figure S22. Comparision of charactiristic region in ^{1}H NMR spectra of rac-nornicotine and *rac*-nornicotine in the presence of naproxen.
